# Computational modelling of the suppression of optic nerve fibre

**DOI:** 10.1007/s11517-026-03541-z

**Published:** 2026-02-23

**Authors:** Ariastity Mega Pratiwi, Orsolya Kekesi, Alejandro Barriga-Rivera, Gregg Suaning

**Affiliations:** 1 Department of Applied Physics III, University of Seville, Seville, Spain; 2 School of Biomedical Engineering, University of Sydney, Sydney, NSW Australia; 3 Freiburg Institute for Advanced Studies, University of Freiburg, Freiburg, Germany

**Keywords:** Visual neuroprosthesis, Computational modelling, Optic nerve, Selective stimulation

## Abstract

Retinal neuroprostheses aim to restore vision in degenerated retina but are hindered by poor activation selectivity. Suppressing subsets of optic nerve fibres based on diameter may improve selectivity by filtering superfluous activations. This study evaluates frequency-induced neuromodulation (FIN) using sinusoidal waveforms to achieve diameter-selective suppression. Computational models of retinal ganglion cells (RGCs) and optic nerve fibres (1.4, 2.8, and 4.3 μm) were subjected to sinusoidal currents from 10 to 10,000 Hz. The range of optic nerve fibre diameters was chosen to represent the optic nerve fibre population in rats. FIN could selectively filter action potential conduction, with the highest suppression probability at frequencies of 1000 Hz and above. The current amplitude required for suppression was influenced by the diameter of the fibres, the sinusoidal frequency of the FIN waveforms, and the ON-OFF typing of the RGC. It was observed that the membrane potential upstream of the FIN application site influenced the response to FIN. Selective suppression based on the fibre diameter is plausible, whereas selective functional-suppression based on the ON-OFF typing is less plausible owing to the small differences in their respective suppression thresholds. Filtering of this kind may improve the utility of retinal neuromodulation as a treatment to blindness.

**Graphical Abstract:**

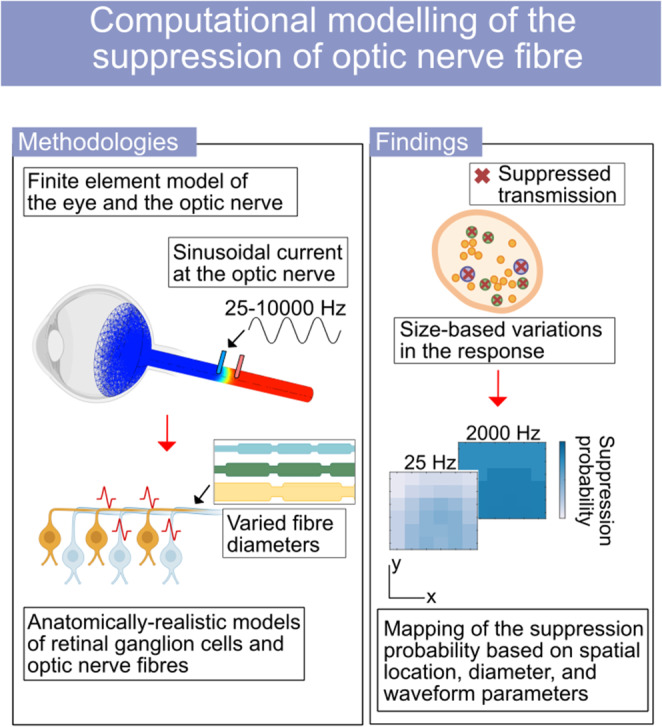

**Supplementary Information:**

The online version contains supplementary material available at 10.1007/s11517-026-03541-z.

## Introduction

Visual neuroprostheses use electric currents to elicit responses from surviving neural cells to create *phosphenes* – visual sensations that ideally appear as distinct and localised perceptions of light. Ongoing research focuses on improving stimulation efficacy to produce selective activation, i.e., only activating a small subset of neurons with a specific function at the intended location. However, the complexity of the retina presents an incompatibility with electrical current stimulations. Lateral spreading of the current can and clearly does cause inadvertent activation alongside the target cells [1]. As neighbouring neurons may codify different visual information [2], unintended activations can increase the complexity of phosphenes.

Strategies suggested to induce selective activation include electrical pulse optimisation at the retina to target a specific neuron type, typically involving the modulation of pulse width [3], amplitude envelope [4], or frequency [5]. Instead of attempting selective stimulations within the retina, we propose that neuromodulation at a secondary site - the optic nerve - can inhibit some neural activation to reduce unwanted activations. Hence, the blocking of neural activations at the optic nerve offers potential benefits to the visual outcomes of visual prosthesis users.

The use of simultaneous modulation was first proposed by Guo et al. [6]. This study expands on the idea by considering the variation of optic nerve fibre diameters. Mammalian optic nerves, including humans’, contain differently sized axon fibres [7, 8]. Association between fibre size and RGC function [9] and spatial segregation of different fibre size groups in the optic tract have been reported [7, 8], suggesting that geometric-filtering based on fibre size in the optic nerve could contribute to improved selectivity in retinal stimulation by blocking transmissions from a subset of neurons with different functions, receptive fields, and terminal locations. For example, the small, intermediate, and large fibres in cats are associated with W, X, and Y-RGCs, respectively. The different types of RGCs are responsible for different functions, with Y cells being sensitive to movement and X cells being sensitive to patterns and fine details. Suppressing one cell type over another, e.g., suppressing Y cells, can focus the activations to fine details, increasing the acuity. In other species, such as rodents, Y-like cells exist [10]. However, the functional association with cell size is not as straightforward as in cats. Recent studies suggested that including other morphological signatures, such as the stratification depth, alongside the dendritic field size would yield a more accurate functional classification of an RGC [11].

Frequency-induced neuromodulation (FIN) has been demonstrated in the peripheral (PNS) and central (CNS) nervous systems to achieve both activation and inhibition [12, 13]. In the CNS, reversible conduction blocking was found to occur at frequencies > 150 Hz, while 25–150 Hz was found to be efficacious for selective activations of retinal cells [14] and hippocampal cells [13]. Computational studies have previously indicated that fibre diameter affects the threshold amplitude required for suppression in the PNS [15, 16]. We seek to explore and ultimately exploit the relationship between axon geometry and frequency-induced suppression to improve the efficacy of visual neuroprostheses.

While the inhibition of PNS fibres is relatively well-known, inhibition of optic nerve conduction is largely unexplored. A notable exception is the investigation of cat’s optic nerve suppression using mechanical pressure [17]. Y-RGCs were suppressed for at least 4 days after the application of pressure, while the response carried by smaller fibres were unchanged. Thus, it was concluded that mechanical pressure selectively inhibited the larger fibres, possibly due to myelin degeneration that could be retained for weeks [18]. As myelin degeneration is not readily reversible, suppression using electric current may offer a more suitable approach.

Here, the feasibility of selective suppression according to fibre diameter and/or, by morphological association, the suppression of certain retinal ganglion cells (RGC) types, namely the ON and OFF types, by applying sinusoidal electrical current to the optic nerve was explored using an in-silico model.

## Methods

The computational modelling method followed the procedure described by McNeal [19]. Briefly, a finite element model (FEM) of retinal tissue and optic nerve based on murine anatomy was used to calculate the electric potential distribution in response to electrical current, which were then applied as extracellular voltage to ON and OFF optic nerve fibre models. Neuronal dynamics were defined using Hodgkin-Huxley-style equations [20]. For details of the described procedure, refer to our previous work [21].

### The FEM model of the retinal tissue and the optic nerve

**Fig. 1:**
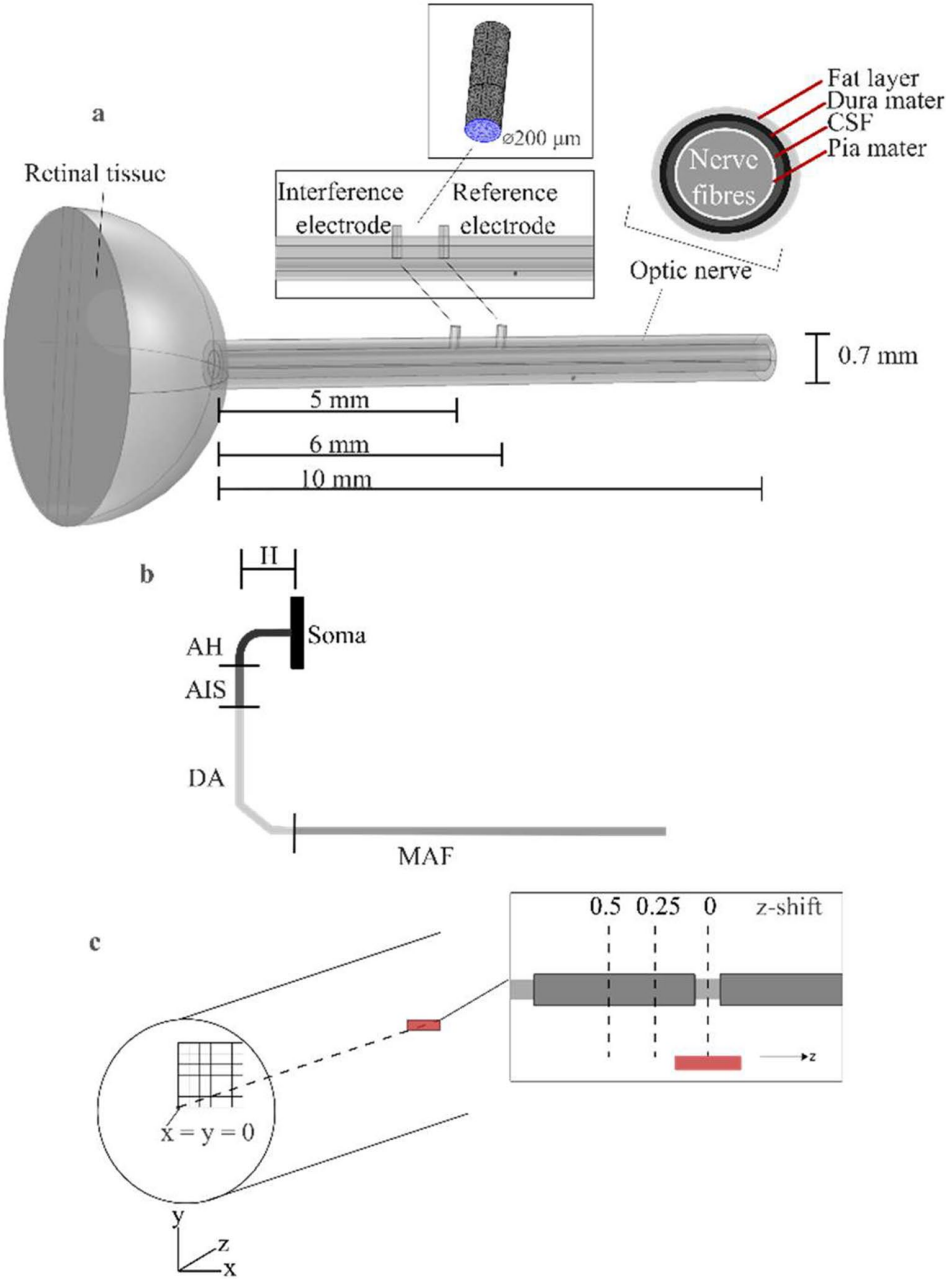
(**a**) The geometry of the eye and optic nerve’s FEM. The optic nerve layers include the fat, dura mater, CSF, pia mater, and nerve fibre space. The locations of the interference and reference FIN electrodes are shown in the cross-section. The electrode geometry is shown in the inset, with the active area highlighted in blue. (**b**) Segmenting the combined RGC-optic nerve fibre model to soma, AH (axonal hillock), AIS (axon initial segment), DA (distal axon), and MAF (myelinated axon fibre). H denotes the height of the axonal section distinguishing ON from OFF RGC. (**c**) The square grids showing the 36 possible locations of the fibre model on the x-y plane. The FIN electrodes were placed on the longitudinal axis of the optic nerve. For each fibre, three z-positions were simulated (z-shift = 0, 0.25, and 0.5)

The FEM model, built using the ‘electric current’ module in COMSOL Multiphysics^®^ 5.6, included a spherical cut of the eye with a single conductivity value for the retina [22] and a cylindrical section with multiple conductivity values to represent the anatomical layers of the optic nerve, such as the pia, cerebrospinal fluid, dura, and fat [23] (Fig. 1). The total length of the optic nerve section was 10 mm long to avoid distortion in electric potential calculation that might be caused by the model’s boundaries being too close to current source [24]. The geometrical parameters and conductivity values of the retina and the layers of the optic nerve were based on pre-existing murine models (Supplementary Information 1).

To deliver the frequency-induced neuromodulation (FIN) current, a pair of planar electrodes were incorporated into the optic nerve model. These electrodes were aligned along the longitudinal (rostrocaudal) axis of the nerve. Each electrode featured a circular conductive surface with a diameter of 200 μm, situated on the midline of the nerve fibre space, which equates to a penetration depth of 283.47 μm (the sum of the thicknesses of the fat layer, dura mater, CSF, pia mater, and the radius of the nerve fibre space. See Supplementary Information 1 for the dimensions used in the model). The electrode positioned more rostrally (5 mm away from the soma of the RGC) was designated as the interference electrode, while the more caudal electrode (6 mm away from the soma of the RGC) was designated as the reference electrode. This configuration created a bipolar stimulation setup, which served to localize the electric field and minimize current spread beyond the targeted region of the optic nerve [25]. To investigate spatially dependent suppression effects, the relative positions of the optic nerve fibres and the electrodes were systematically varied along the x, y, and z axes, allowing the assessment of the effects of electrode–fibre geometry on the likelihood of suppression.

The conductive surface of the interference electrode was modelled as a current source (Neumann boundary condition), where the sinusoidal FIN current amplitude is defined. In contrast, the reference electrode was modelled as a ground (Dirichlet boundary condition) with a 0 V potential, to act as a current sink. Note that we did not explicitly model the conductivity of the electrode material (e.g., platinum). Rather, the conductivity of the nerve fibre space was applied to the electrode. This was because the focus of the study is on the effects of FIN on the optic nerve fibre’s activations, rather than to optimise the design of the electrode itself. Importantly, this conductivity choice does not affect the electric field at the interface between the electrode’s active surface and the tissue, owing to the use of current boundary conditions to represent an idealised current source. While it may influence the electric field around the non-active regions of the electrode, this effect lies outside the scope of the present work.

Extracellular potentials generated by the bipolar electrode configuration were first computed in COMSOL as three-dimensional potential maps. These maps were exported and interpolated onto the midpoint of each neuronal compartment in NEURON environment version 8.2 [26] using linear interpolation. At each integration timestep, the interpolated potential was applied as an extracellular voltage to the corresponding compartment.

Two types of combined retinal ganglion cell (RGC)–optic nerve fibre models were developed to represent ON and OFF cell types. Each model included the soma, dendrites, axon hillock (AH), axon initial segment (AIS), unmyelinated distal axon (DA), and the myelinated axon fibre (MAF) section (Fig. 1b). To assess spatial variability in response to FIN, the fibre models were positioned at different locations within the optic nerve. In the transverse (x–y) plane, a 200 × 200 μm quadrant of the optic nerve cross-section (upper right quadrant) was divided into 36 grid cells (each 40 × 40 μm), with one fibre placed at the centre of each grid cell (Fig. 1c). Only one quadrant was simulated due to the symmetry of the FEM geometry.

The model also accounts for the variations in the longitudinal axis between the electrodes and the fibre nodes. In the longitudinal (z) direction, each fibre’s position was varied using three z-shift values, defined as fractions of the node-to-node distance (z-shift = 0, 0.25, and 0.5). These shifts altered the vertical alignment between the electrode centre and the nearest node of the fibre, thereby changing the Euclidean distance between them and enabling analysis of z-axis sensitivity to FIN. At z-shift = 0, the centre of the interference electrode is vertically aligned with the centre of the nearest node, while at z-shift = 0.5, the interference electrode is positioned halfway between the two nodes of a fibre. Note that due to the variations in the internodal distance with the fibre diameter, the same z-shift value would result in different longitudinal distances between the electrode and the node for different fibre diameters.

### The combined RGC-optic nerve model

The three-dimensional morphology of the RGC model, spanning from the dendritic field to the distal axon (DA), was adapted from a mouse RGC template [27, 28], with the dendritic field diameter rescaled to 400 μm to match the dimensions of a rat α-RGC [29]. The morphologies of the ON and OFF fibre models were differentiated by the vertical distance between the AH and the soma, which was 40 μm longer in the OFF model than in the ON model [30]. All other morphological features, including the soma and dendritic field, were kept identical between the two models.

The biophysical dynamics from the dendrites to the DA were based on the model by Guo et al. [31, 32], with modifications to ion channel conductance’s using a Markov Chain Monte Carlo (MCMC) approach [33] with a Metropolis-Hastings algorithm [34, 35]. These adjustments were made to better fit electrophysiological data from rat RGCs [29, 36]. Detailed morphological and electrical parameters (excluding the myelinated axon fibre, MAF) are provided in Supplementary Information 2, while the conductance fitting procedure and validation results are described in Supplementary Information 3.

**Fig. 2:**
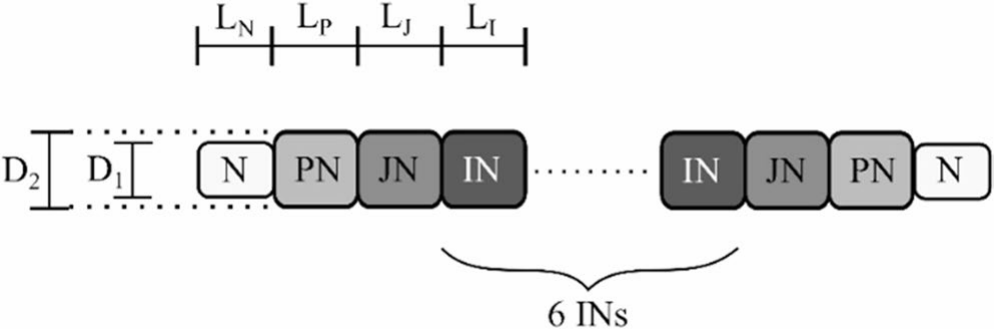
The division of optic nerve fibre morphology. N = nodal, PN = paranotal, JN = juxtaparanodal, and IN = internodal compartments. Each node-to-node unit contained 2 PNs, 2 JNs, and 6 Ins D1 and D2 are the nodal diameter and the fibre diameter, respectively. L denotes the length of each compartment

Both ON and OFF models included only myelinated axons, consistent with the fact that extraocular optic nerve fibres are myelinated in most mammals [37]. The MAF section was implemented using the McIntyre-Richardson-Grill (MRG) double cable model [38], which segments the axon into distinct regions and applies myelin sheaths to all but the nodal compartments (Fig. 2).

Three fibre diameters ($\:{d}_{f}$) were modelled, which are 1.4, 2.8, and 4.3 μm. These values correspond to the modal diameters of the three primary fibre groups observed in the rat optic nerve, as reported by de Juan et al. [39]. To reflect the natural distribution of fibre sizes, each diameter was assigned a probability weight: 0.903 for 1.4 μm, 0.094 for 2.8 μm, and 0.003 for 4.3 μm. Myelin thickness and segment lengths for each diameter were interpolated from the data provided by Li et al. [40]. Ion channel kinetics were adopted from established mammalian optic nerve fibre models [40, 41], with the assumption that ion exchange occurs predominantly at the nodes of Ranvier. A complete list of geometrical and electrical parameters used for the myelinated axon fibre (MAF) sections is provided in Supplementary Information 4.

## Simulation parameters

To evaluate the efficacy of frequency-induced neuromodulation (FIN), simulations were conducted using the fitted ON and OFF RGC–optic nerve fibre models. A continuous spike train was generated in each model by injecting a constant intracellular current into the soma. This approach was chosen to isolate the effects of FIN on the optic nerve and avoid confounding interactions (e.g., current superposition or crosstalk) between the retinal stimulation site and the FIN electrodes [42].

A monophasic, constant intracellular current of 0.13 nA was applied for 40 ms (activating stimulus I_stim_). This current amplitude and duration used was sufficient to maintain consistent spiking throughout the 50 ms simulation window. This stimulation was not intended to replicate realistic extracellular stimulation parameters but rather to serve as a controlled method for generating action potentials for downstream suppression analysis. The same amplitude of I_stim_ was used for all fibre diameters and types. Although ON and OFF RGCs have different excitability due to morphological and biophysical differences, preliminary testing showed that all models produced stable spike trains under this condition. This uniform stimulation allowed for consistent comparison of FIN effects across fibre types.

Simultaneously, extracellular FIN was delivered via the bipolar electrodes at the optic nerve. FIN amplitudes ($\:{\mathrm{I}}_{\mathrm{I}\mathrm{n}\mathrm{t}}$) ranged from 0 to 400 µA in 40 µA increments. Frequencies tested (f_int_) included 10, 25, 50, 100, 250, 350, 450, 500, 550, 650, 750, 1000, and then in 1000 Hz steps up to 10,000 Hz. This range was selected to encompass both low-frequency regimes previously shown to modulate CNS activity (25–150 Hz) and high-frequency regimes (≥ 1 kHz) associated with conduction block and differential RGC responses [14, 43, 44].

All simulations were implemented in NEURON with custom differential equations for the ion channel mechanisms written in NMODL files. The equations are numerically solved using the backward Euler approximation with the integration timestep fixed at 0.01 ms (tenfold smaller than the shortest FIN period at 10 kHz) to ensure adequate temporal resolution. Each simulation was run for 50 ms, and spike counts were recorded at multiple nodes along the optic nerve. A spike was defined as any transmembrane potential crossing 0 mV to filter out subthreshold oscillations induced by the sinusoidal FIN waveform.

## Results

### Suppressions occurred due to FIN applications

**Fig. 3:**
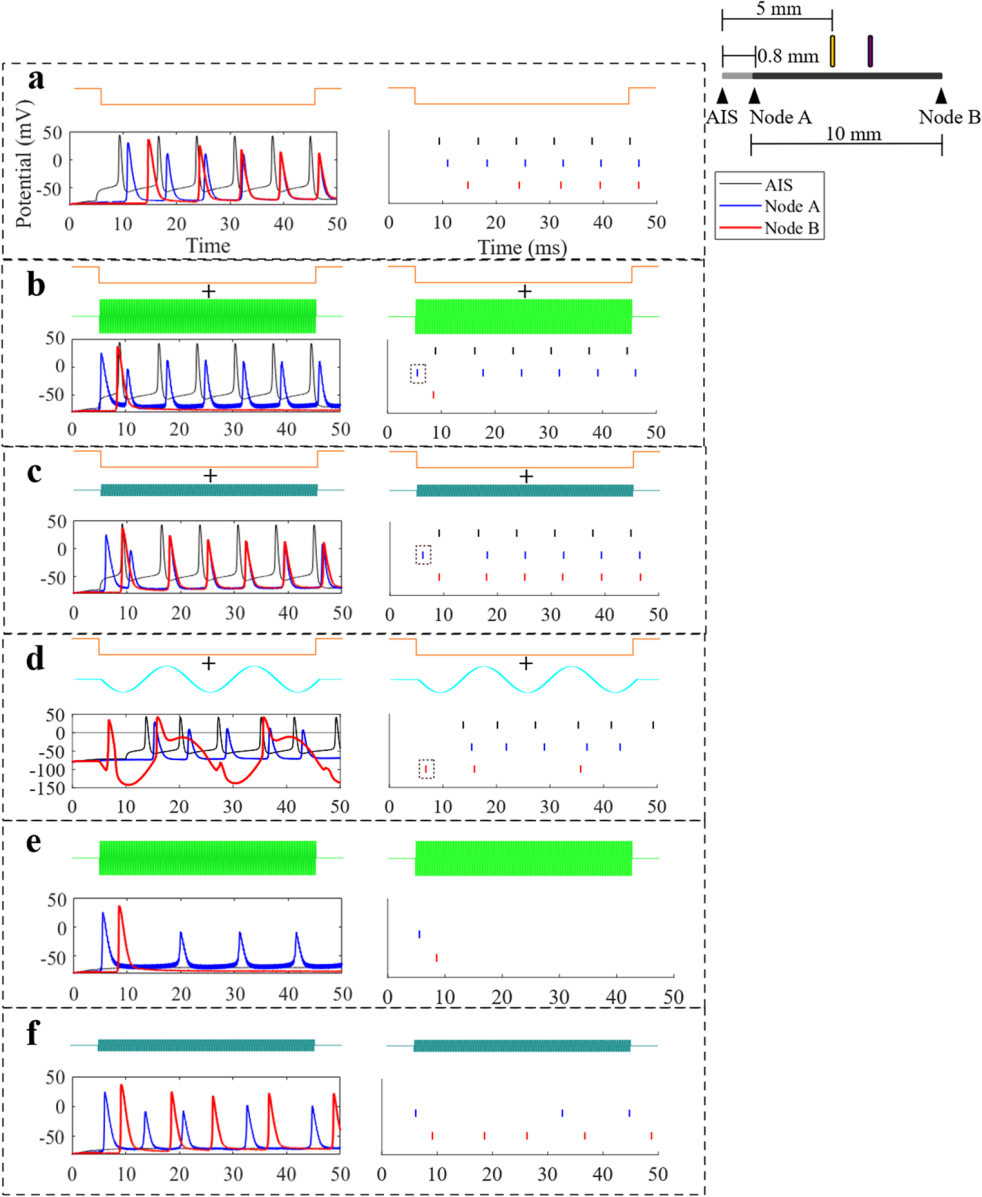
(**a**) Only $\:{I}_{Stim}$ was delivered. All spikes from the AIS travelled to node B. (**b**) Complete suppression occurred ($\:{I}_{Int}$ = 300 µA, $\:{f}_{Int}$ = 4000 Hz) and one spike was detected at node B. Node A’s first spike preceded AIS’ first spike, demonstrating that the spike was caused by FIN. (**c**) Excitation ($\:{I}_{Int}$ = 80 µA, $\:{f}_{Int}$ = 4000 Hz) produced an additional spike, besides enabling the conduction of spikes from the AIS to node B. (**d**) Partial suppression ($\:{I}_{Int}$ = 40 µA, $\:{f}_{Int}$ = 50 Hz) involved eliciting a spike in the optic nerve while simultaneously suppressing some of the AIS spikes. The temporal pattern of the spiking at the AIS was also altered due to the direct modulation of the AIS’ membrane by FIN. (**e**) FIN ($\:{I}_{Int}$ = 300 µA, $\:{f}_{Int}$ = 4000 Hz) applied alone without $\:{I}_{Stim}$. One suprathreshold spike was detected both at nodes A and B. (**f**) FIN ($\:{I}_{Int}$ = 80 µA, $\:{f}_{Int}$ = 4000 Hz) applied alone without $\:{I}_{Stim}$. Lower $\:{I}_{Int}$ resulted in multiple suprathreshold spikes to be detected at nodes A and B

Applying FIN together with retinal stimulation ($\:{I}_{Stim}$) at various frequencies and amplitudes resulted in different phenomena. These effects can be categorised into three types, which are partial suppression, maximal suppression, and excitation. To illustrate each case, the transmembrane potentials for ON fibre at $\:{d}_{f}$ = 1.4 μm were examined at three key recording locations, which are the centre of AIS, the most proximal node (node A, located 4.2 mm proximal to the interference electrode), and the most distal node (node B, located 5 mm distal to the interference electrode) (Fig. 3). The number of the detected spikes at node B is called $\:{n}_{spike}$, and it was used to quantify the degree of suppression.

Figure 3a shows the transmembrane potentials at the baseline condition, where no FIN was delivered and only $\:{I}_{Stim}$ was present. Under this condition, a spike train consisting of 6 action potentials within 50 ms travelled from the AIS towards nodes A and B, demonstrating the expected temporal delays. When FIN was applied at high frequency ($\:{f}_{Int}$= 4000 Hz and $\:{I}_{Int}\:$= 300 µA), a maximal suppression occurred, suppressing the transmission of all the spikes from the AIS towards node B (Fig. 3b). In the case of maximal suppression, $\:{n}_{spike}$ at node B is only 1. Even when a train of action potentials were detected at node A, none reached node B. However, a single additional spike was generated at nodes A and B, preceding the first spike at AIS, suggesting that the additional spike was caused by the FIN itself, prior to suppression.

Lowering $\:{I}_{Int}$while maintaining the $\:{f}_{Int}$ ($\:{f}_{Int}$ = 4000 Hz and $\:{I}_{Int}$ = 80 µA) resulted in an excitation that was not accompanied by suppression (Fig. 3c). In this case, there was an increase in the $\:{n}_{spike}$ compared to the baseline condition. This is due to the additional single spike generated by FIN and the failure of suppression due to the subthreshold $\:{I}_{Int}$.

Using a lower frequency FIN ($\:{f}_{Int}$ = 50 Hz and $\:{I}_{Int}$ = 40 µA) resulted in a partial suppression, where multiple spikes were detected at the final node, but still reduced compared to the baseline. Some spikes from the AIS were blocked, while others were generated at the distal node. This likely resulted from the high charge per phase contained within the FIN waveform, which produced direct modulation of the distal node and the AIS, as suggested by the altered spike timings at the AIS (Fig. 3d). This could be due to the prolonged hyperpolarisation caused by FIN’s anodal phase, creating an anodal block.

To isolate FIN’s contribution, the waveform was applied without retinal stimulation. At high amplitudes, FIN alone evoked a single suprathreshold spike at nodes A and B, plus subthreshold depolarizations at node A (Fig. 3e), confirming the initial spike originated from FIN. At lower amplitudes, FIN produced multiple suprathreshold spikes at both nodes (Fig. 3f), showing direct optic nerve activation. However, fewer spikes occurred than when FIN was paired with $\:{I}_{Stim}$, indicating that increases in $\:{n}_{spike}$ at node B under subthreshold FIN reflect both FIN-driven and AIS-evoked activity.

### The frequency response of optic nerve fibres with varying diameter

**Fig. 4:**
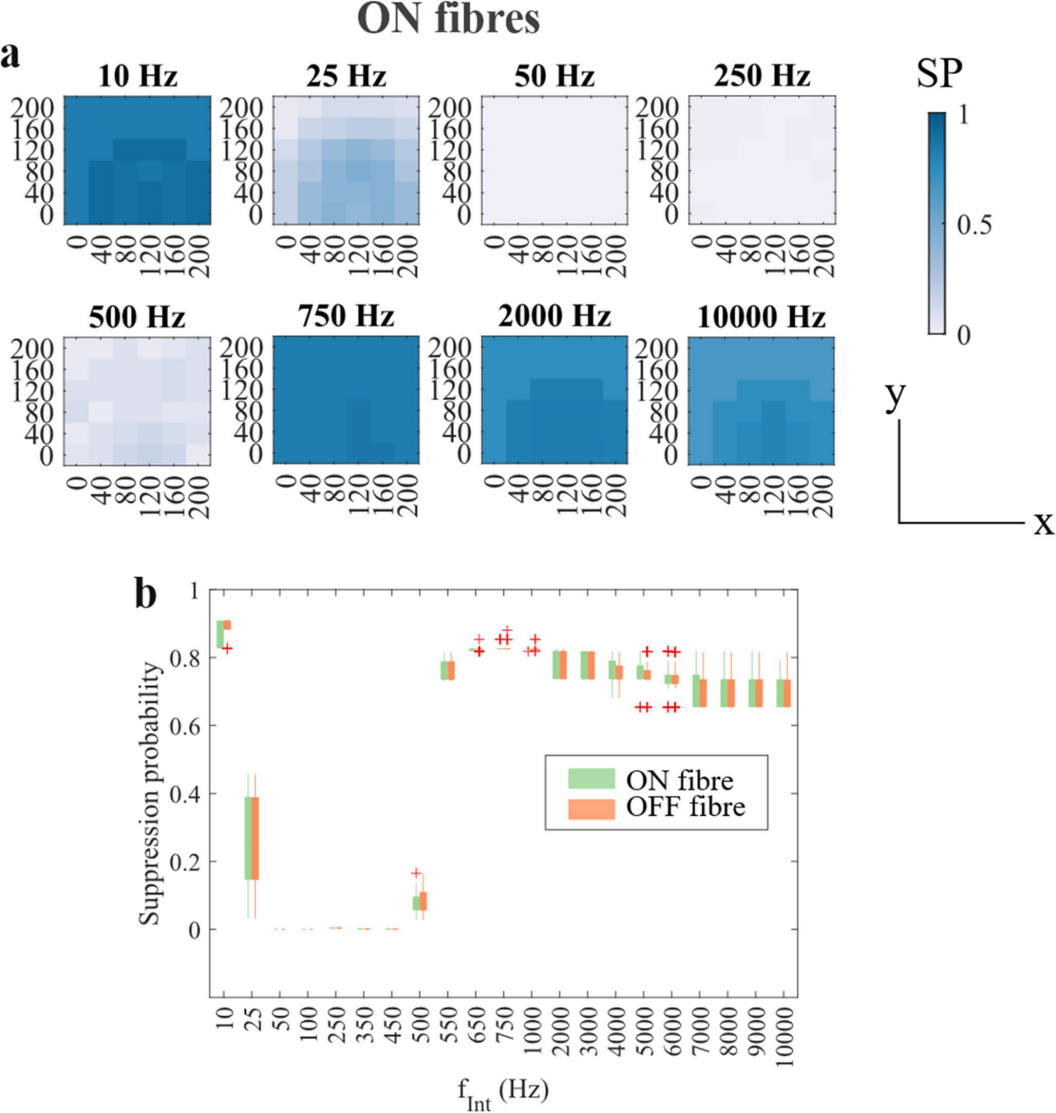
(**a**) Colour grids showing the suppression probability (SP) produced by all combinations of $\:{I}_{Int}$ and $\:{f}_{Int}$ for ON fibres at different x-y positions. Each grid represents the probability that a fibre in that location would be maximally suppressed ($\:{n}_{spike}$ ≤ 1). Dark blue indicates the highest SP of 1 (all the tested combinations of $\:{I}_{Int}$ and $\:{f}_{Int}$ maximally suppressed the action potential transmission), while white indicates the lowest SP of 0 (none of the tested combinations of $\:{I}_{Int}$ and $\:{f}_{Int}$ produced maximal suppression). (**b**) The boxplots comparing the SP of the ON and OFF fibres, including all fibre diameters and in all x-y positions

While the suppressive effect of FIN has been illustrated in the previous section by using the spike count at the most distal node ($\:{n}_{spike}$), the contribution of each diameter and z-shift can be represented by the Suppression Probability (SP). The colour maps in Fig. 4 represent the SP for each x-y location at different $\:{f}_{Int}$. For each grid in a map of a given $\:{f}_{Int}$, $\:{SP}_{x,y,\:fint}$ was calculated following Eq. 1 below:

$$\:{S}_{x,y,fInt}=\left\{{F}_{z=k,d=m}\right\}$$

$$\:{s}_{x,y,fInt}=\left\{F|{n}_{spike}\:of\:F\le\:1\right\}$$

$$\:{SP}_{x,y,\:fInt}=\:\frac{{\sum\:}_{I=c}^{}(n\left(s\right)\times\:p\left(s\right))}{{\sum\:}_{\:I=c}^{}\left(n\right(S)\times\:p(S\left)\right)}$$

$$\:\:\:\:\:\:\:\:\:\:\:\:\:k=\left\{0,\:0.25,\:0.5\right\};m=\left\{1.4,\:2.8,\:4.3\:\mu\:m\right\};c=\left\{0,\:40,\dots\:,\:400\:\mu\:A\right\}\:\:\:\:\:\left(1\right)$$

Here, $\:{S}_{x,y,fInt}$ represents a set of all fibres simulated in given x and y positions, at that particular $\:{f}_{Int}$, while $\:F$ refers to a particular fibre model. In each grid, the results from all three diameters and all three z-shifts were incorporated (an analysis on the effects of the z-shift is presented in Supplementary Information 5). In contrast, $\:{s}_{x,y,fInt}$ represents a subset of $\:{S}_{x,y,fInt}$, whose detected $\:{n}_{spike}$ was equal to or less than 1 (maximally suppressed). The probability weight of the fibre was represented by $\:p\left(x\right)$, which was defined for each fibre diameter. Hence, SP indicates the likelihood of the fibre located in a particular x-y-location to be maximally supressed when FIN is used.

For example, the SP at x = y = 0, with $\:{f}_{Int}$ = 10,000 Hz is 0.654. To acquire this value, the parameters that resulted in maximal suppression were rounded up. At $\:{d}_{f}$ = 1.4 μm, 7 out of 11 available current amplitudes ($\:{I}_{Int}$ ≥ 160 µA) produced maximal suppression for all three z-shifts, while at $\:{d}_{f}$ = 2.8 and 4.3 μm, 9 out of 11 available current amplitudes ($\:{I}_{Int}$ ≥ 80 µA) produced maximal suppression for all three z-shifts. Thus, the SP was calculated as:

$$\:SP=\frac{\left(7\times\:3\times\:0.903\right)+\left(9\times\:3\times\:0.094\right)+(9\times\:3\times\:0.003)}{\left(11\times\:3\times\:0.903\right)+\left(11\times\:3\times\:0.094\right)+\left(11\times\:3\times\:0.003\right)}=0.654$$

In most frequencies, the x-y-location of the nerve fibre (Fig. 4a) influenced the SP values, although the variations in the SP values were not more than 0.02 across all FIN frequencies. The difference in the fibre location resulted in the difference of electric potential experienced by the membranes, which was likely to alter the membrane response to FIN.

Additionally, SP was strongly influenced by FIN frequency (Fig. 4b). A non-linear trend in the SP value relative to the frequency of FIN for both fibre types was observed. At 10 Hz, both ON and OFF fibres exhibited high suppression, which decreased at 25 Hz and further at 50 Hz. Between 50 and 450 Hz, suppression was largely absent. However, SP began to rise again from 500 Hz, peaking around 750 Hz, and remained relatively stable at higher frequencies up to 10,000 Hz.

SP values slightly varied between the ON and OFF fibres at some frequencies. A statistically significant difference between ON and OFF fibres was observed only at 10 Hz (unpaired t-test, *p* < 0.05), with a non-significant but notable difference at 650 Hz (*p* = 0.33). In most cases, the trends shown by the ON and OFF fibres did not differ significantly.

**Fig. 5:**
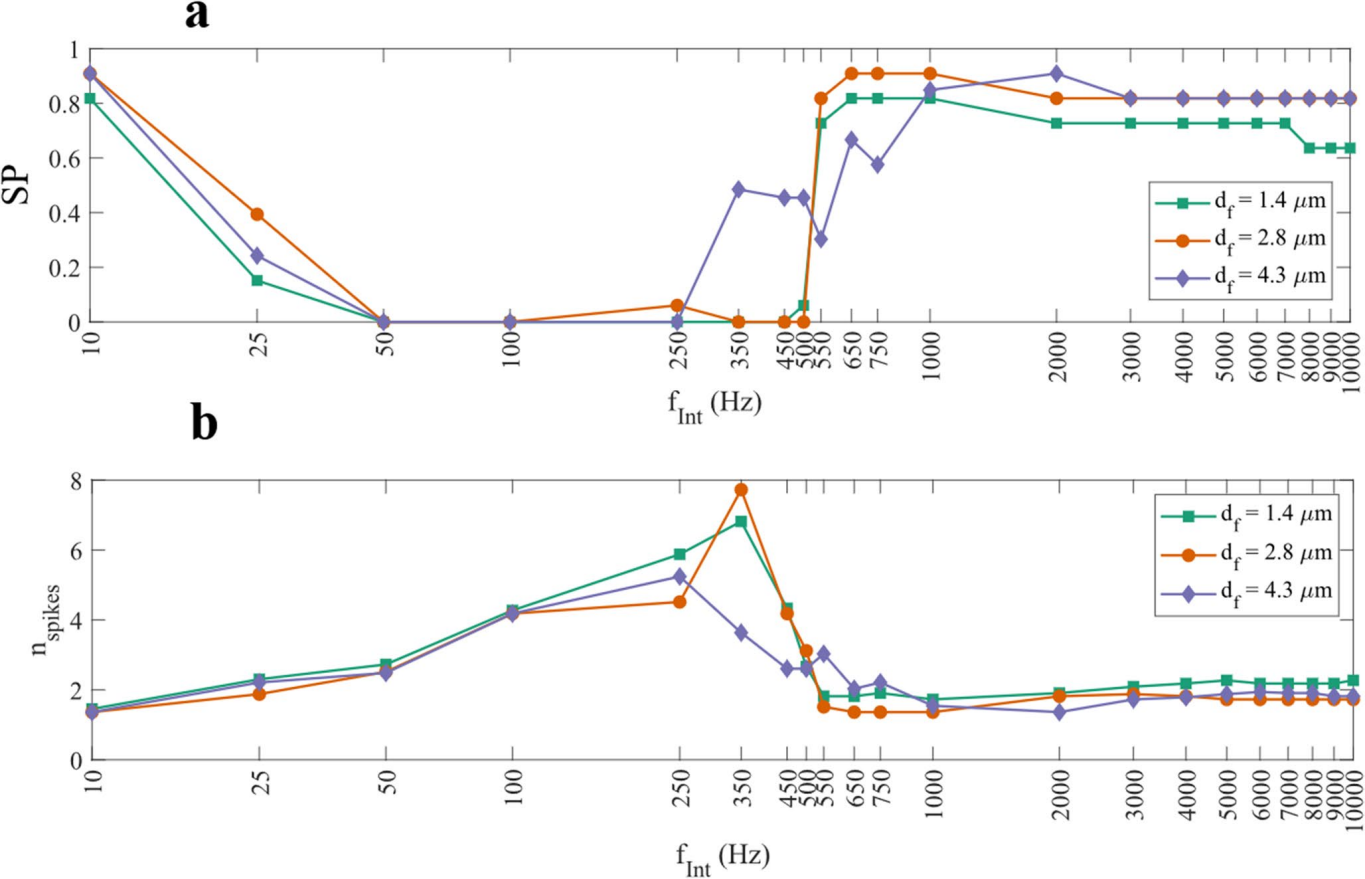
(**a**) The SP plotted against $\:{f}_{Int}$ for each fibre diameter ($\:{d}_{f}$) for the ON fibres. Variations of the SP between different fibre diameters were seen in all frequencies, except for $\:{f}_{Int}$ = 50–100 Hz. In almost all frequencies, the smallest $\:{d}_{f}$ was suppressed the least. (**b**) The number of spikes against $\:{f}_{Int}$ plotted as stacked bar charts for ON fibres at different $\:{d}_{f}$. At all frequencies, the $\:{n}_{spike}$ at the last node differed significantly based on the fibre diameter $\:{d}_{f}$ (ANOVA, *p* < 0.05)

To investigate how FIN affects fibres of different diameters, SP was plotted against $\:{f}_{Int}$​ for each diameter group (Fig. 5a). Each data point represents the aggregated SP across all spatial positions (x, y, z) for fibres of a given diameter. The analysis revealed that both fibre diameter and $\:{f}_{Int}$ significantly influenced suppression outcomes for optic nerve fibres (ANOVA, *p* < 0.05). The results are similar for both ON and OFF fibres, with fibre diameters and $\:{f}_{Int}$ significantly influencing $\:{n}_{spike}$ (ANOVA, *p* < 0.05 in Fig. 5b).

At lower frequencies (< 1000 Hz), suppression was highly variable and exhibited non-linear trends. At 10 Hz, SP was high across all fibre types and diameters, but SP dropped sharply at 25 Hz and further at 50 Hz, indicating the reduced suppression. Between 50 and 450 Hz, maximal suppression was not found for any fibre type, diameter, or location, suggesting that this range may fall below the threshold required for suppression to occur.

Increasing the frequency further to the intermediate frequency range (550–750 Hz) caused SP to re-emerge in a fibre size-dependent manner. In contrast to trends observed at higher frequencies, the largest fibres ($\:{d}_{f}$ = 4.3 μm) were suppressed the least in this range, while smaller fibres showed greater susceptibility. At other frequencies where maximal suppression happened, the smallest fibre ($\:{d}_{f}$ = 1.4 μm) was suppressed the least. This reversal suggests a transitional regime where the suppression mechanism may be shifting from one to another.

## Differential effects of FIN at low, intermediate, and high frequencies

**Fig. 6:**
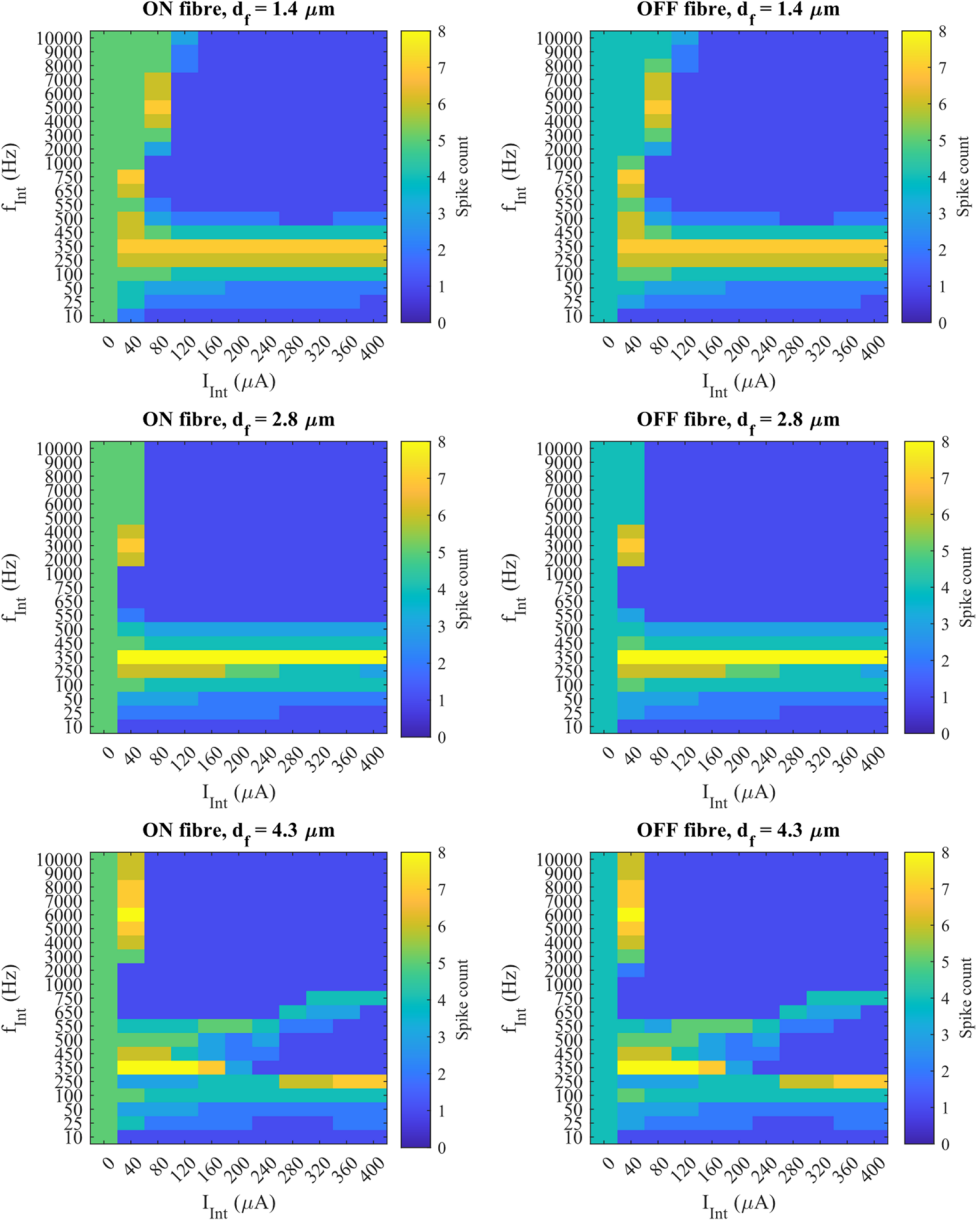
The colour maps of $\:{n}_{spike}$ for ON and OFF fibres at the base position. A threshold FIN amplitude, $\:{th}_{Sup}$, could be identified for each $\:{f}_{Int}$ ≥ 750 Hz for $\:{d}_{f}=$ 1.4 and 2.8 μm, and $\:{f}_{Int}$ ≥ 1000 Hz for $\:{d}_{f}=$ 4.3 μm

As previously shown, suppression probability (SP) varied nonlinearly with FIN frequency, suggesting that different mechanisms may dominate across distinct frequency ranges. To explore these mechanisms, we examined the number of action potentials ($\:{n}_{spike}$​) detected at the most distal node of ON and OFF fibres located at the base position (x = y = 0 μm, z = 0) relative to the FIN interference electrode. Figure 6 illustrates the change in $\:{n}_{spike}$ with $\:{I}_{Int}$ and $\:{f}_{Int}$.

At lower frequencies (< 1000 Hz), suppression was generally partial. Although $\:{n}_{spike}$​ was reduced compared to baseline (i.e., when $\:{I}_{Int}$ = 0 µA), more than 1 spike were detected at the most distal node and maximal suppression was rarely achieved. In many cases, spikes reappeared at higher amplitudes, indicating that suppression was not stable. This partial suppression likely reflects the inability of low-frequency FIN to maintain consistent hyperpolarisation or disrupt spike propagation effectively. Notably, even when suppression occurred, more than one spike was often still detected, confirming that the effect was incomplete.

In contrast, suppressions happening at the higher frequency range ($\:{f}_{Int}$ ≥ 1000 Hz) were more robust and consistent, and maximal suppression was likely. A suppression threshold amplitude ($\:{th}_{Sup}$) could be identified for all the tested $\:{d}_{f}$, beyond which maximal suppression was maintained regardless of further increases in amplitude. For $\:{d}_{f}$ = 1.4 and 2.8 μm, $\:{th}_{Sup}$ was found for $\:{f}_{Int}$ > 500 Hz, while for $\:{d}_{f}$ = 4.3 μm, $\:{th}_{Sup}$ was found for $\:{f}_{Int}$ > 750 Hz. For the largest fibre diameter, suppressions were less stable in the intermediate range of $\:{f}_{Int}$ between 500 and 750 Hz, with spikes reappearing at $\:{f}_{Int}$ = 650 Hz. This finding also reflected the result in Fig. 5a-b, where $\:{d}_{f}$ = 4.3 μm was suppressed the least in this frequency range.

**Fig. 7:**
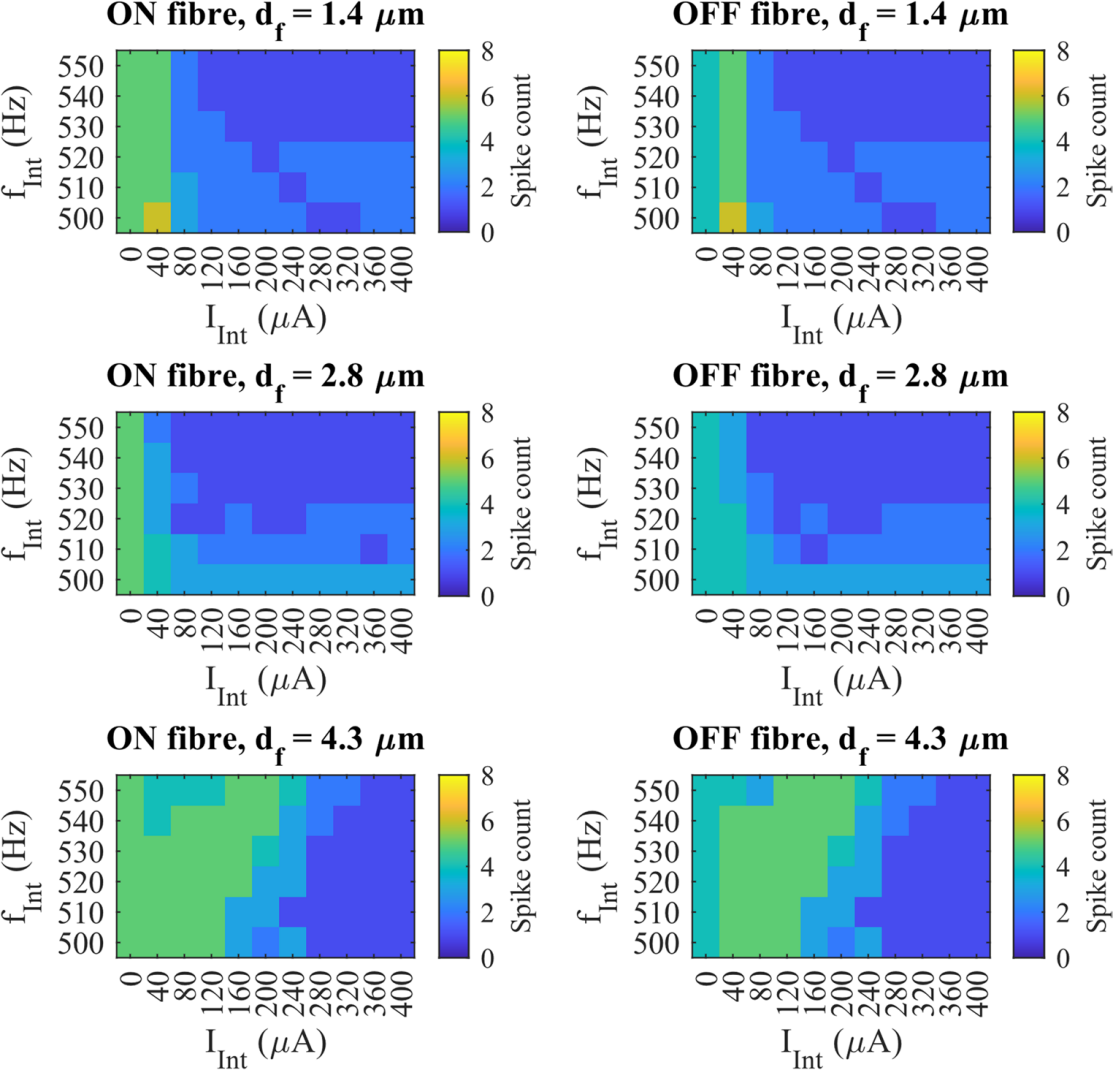
The colour maps showing $\:{\mathrm{n}}_{\mathrm{s}\mathrm{p}\mathrm{i}\mathrm{k}\mathrm{e}}$ at every combination of $\:{\mathrm{I}}_{\mathrm{I}\mathrm{n}\mathrm{t}}$ and $\:{\mathrm{f}}_{\mathrm{I}\mathrm{n}\mathrm{t}}$ for ON and OFF fibre at all tested $\:{\mathrm{d}}_{\mathrm{f}}$, at one x-y location (location 0) with z-shift = 0, for $\:{\mathrm{f}}_{\mathrm{I}\mathrm{n}\mathrm{t}}$ = 500 up to 550 Hz

To better resolve the transition zone between partial and maximal suppression, a finer frequency sweep (10 Hz steps) between 500 and 550 Hz was performed for the fibres at the same spatial location (Fig. 7). For $\:{d}_{f}$ = 1.4 and 2.8 μm, maximal suppression began at 530 Hz, but the $\:{th}_{Sup}$ differed: 160 µA for $\:{d}_{f}$ = 1.4 μm and 120 µA for $\:{d}_{f}$ = 2.8 μm. Increasing the diameter to 4.3 μm did not reduce the $\:{th}_{Sup}$ further. Instead, maximal suppression only started happening at 280 µA at 530 Hz, and maximal suppression was already found at $\:{f}_{Int}$ = 500 Hz, unlike the smaller fibres. These observations suggested that at the intermediate frequency range, the effect of diameter on suppression was reversed as the diameter was increased further. Increasing the diameter could lower the frequency and amplitude threshold for maximal suppression, but also made it easier for spikes to reappear. These competing effects resulted in a non-linear relationship between fibre diameter and suppression threshold, making it less suitable for selective suppression in clinical use.

Note that excitation was observed in multiple frequencies at all fibre diameters, marked by an increase in $\:{n}_{spike}$ relative to the baseline condition. This was observed even at $\:{f}_{Int}$ that was successful in completely suppressing fibre conductions. For example, an excitation occurred at $\:{f}_{Int}$ = 4000 Hz and $\:{I}_{Int}$ = 80 µA for ON fibre with $\:{d}_{f}$ = 1.4 μm just before reaching the $\:{th}_{Sup}$ for maximal suppression. This suggests that FIN can enhance excitability before inducing suppression. Note also that there was always one spike detected in any instance of maximal suppression.

The variation in $\:{n}_{spike}$ compared to the baseline conditions indicated that FIN induced a range of frequency- and amplitude-dependent effects on optic nerve fibres.

These findings help explain the trends observed in Figs. 4 and 5. At 10 Hz, continuous maximal suppression was shown by all fibre models because the half-period of the stimulus waveform matched that of the stimulus duration (50 ms), thus a continuous hyperpolarisation was found. Increasing the frequency up to the intermediate frequency range (around 500 Hz) made the anodal block less effective due to the lower charge per cycle and shorter hyperpolarisation duration, while it might still cause excitations at the distal node. Therefore, FIN at this frequency range mostly resulted in partial suppression or excitations.

## Selective suppressions between ON and OFF fibres were only achievable within a small amplitude window

To evaluate the potential for selectively suppressing either ON or OFF fibres using FIN, the amplitude resolution near the previously identified suppression thresholds ($\:{th}_{sup}$) was refined to 5 µA increments. This finer resolution was applied for ON and OFF fibres across all fibre diameters tested.

**Fig. 8:**
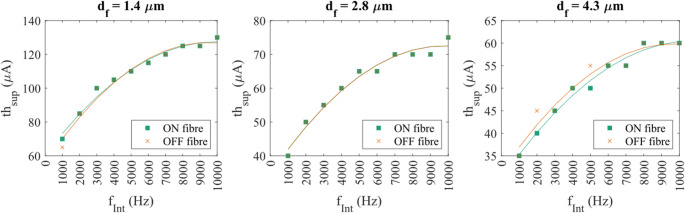
The suppression threshold $\:{th}_{sup}$ for ON and OFF fibres at all $\:{d}_{f}$ tested, for $\:{f}_{Int}$ ≥ 1000 Hz at the base location (x = y = 0 μm, z-shift = 0). Small variations in the $\:{th}_{sup}$ were found between ON and OFF fibre models of the same $\:{d}_{f}$, but it most often occurred at $\:{d}_{f}$ = 4.3 μm (between $\:{f}_{Int}$ = 2000 and 5000 Hz)

Small but measurable differences in $\:{th}_{sup}$ between ON and OFF fibres were observed at specific frequencies (Fig. 8). At $\:{f}_{Int}$ = 1000 Hz and $\:{d}_{f}$ = 1.4 μm, the OFF fibre exhibited a $\:{th}_{sup}$ of 65 µA, or 5 µA lower than the ON fibre at the same diameter. No difference was observed for $\:{d}_{f}$ = 2.8 μm. At higher frequencies ($\:{f}_{Int}$ = 2000 and 5000 Hz), the trend was reversed for $\:{d}_{f}$ = 4.3 μm. Here, OFF fibre required a higher current to be maximally suppressed compared to the ON fibre by 5 µA.

Although $\:{th}_{sup}$ generally increased with frequency for all fibre types and diameters, the amplitude window within which selective suppression could be achieved remained narrow, typically no more than 5 µA. This narrow margin suggests that while selective suppression is theoretically possible, it would be difficult to achieve reliably in practice. Moreover, the lack of consistent directional differences between ON and OFF fibres across frequencies and diameters further limits the feasibility of functional selectivity using FIN alone. These findings also support the conclusion that FIN-induced suppression is primarily governed by frequency and amplitude, rather than total charge delivered.

### Upstream responses cause some differential suppressions in ON and OFF fibres

As the ON and OFF fibre models shared identical morphological and biophysical formulations for the MAF section, any observed difference in the $\:{th}_{sup}$ between ON and OFF fibres are likely attributed to the variations in the transmembrane potentials upstream of the MAF section, namely at the soma, AH, AIS, and the DA section.

As shown in Fig. 5, under the baseline condition when FIN was absent, the ON fibres already fired more action potential spikes than the OFF fibres across all tested $\:{d}_{f}$ when stimulated with the same amplitude of $\:{I}_{Stim}$. As the spikes were initiated in the proximity of the soma, this observation suggests that the cell body of the ON fibre has higher sensitivity to electrical stimuli and therefore a lower activation threshold compared to the cell body of the OFF fibres. This difference in cell body’s excitability could influence how each fibre model responds to FIN, and particularly in terms of the susceptibility to suppression.

**Fig. 9:**
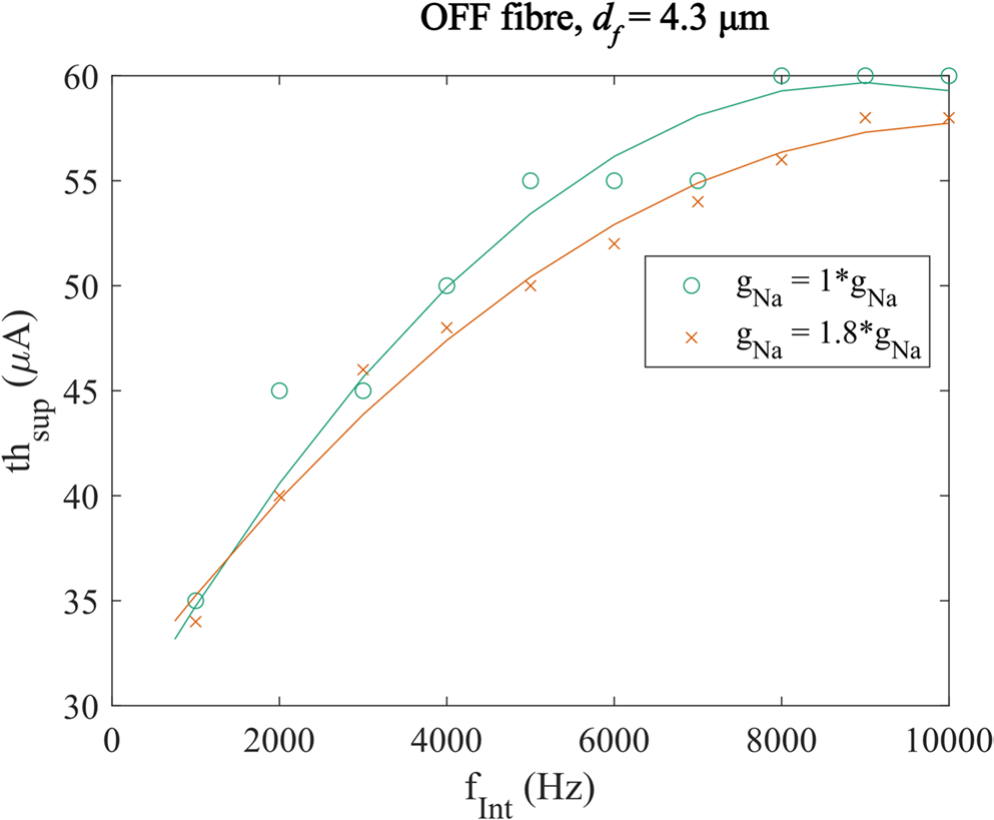
The suppression threshold $\:{th}_{sup}$ for OFF fibre with $\:{d}_{f}$ = 4.3 μm at $\:{f}_{Int}$ = 750–10,000 Hz demonstrated that modifying the sodium channel’s maximum conductance $\:{g}_{Na}$ at the AIS changed the thsup using FIN. At 750 Hz, increasing $\:{g}_{Na}$ increased the $\:{th}_{sup}$, while at higher $\:{f}_{Int}$ an increase in $\:{g}_{Na}$ caused a decrease in $\:{th}_{sup}$, apart from $\:{f}_{Int}$ = 3000 Hz

To test this hypothesis, the density of the sodium channels in and around the cell body of the fibre model were modified to raise its excitability and its $\:{th}_{sup}$ re-measured. Specifically, the maximum sodium channel density of the OFF fibre ($\:{d}_{f}$ = 4.3 μm) at the AIS was increased by a factor of 1.8. The results confirmed that changing the excitability via the modification of sodium channel conductance at the AIS affected the $\:{th}_{sup}$ significantly (Fig. 9). This alteration of suppression threshold was found in all frequencies of FIN of 1000 Hz and above, where the direct modulation of the cell body by AIS was minimal. Higher maximal conductance, and thus increased excitability, decreased the $\:{th}_{sup}$, which further supported the hypothesis that a higher excitability facilitates suppression, but also makes spikes reappearance more likely at higher current amplitudes. For the OFF fibre at $\:{d}_{f}$ = 4.3 μm, the alteration in the thresholds ranged from 1 to 5 µA. However, the threshold gap did not increase linearly with the $\:{f}_{Int}$.

These findings suggest that upstream membrane properties, particularly those governing spike initiation, can influence the fibre’s susceptibility to FIN-induced suppression. Therefore, even subtle differences in ion channel distribution or excitability near the soma and AIS may contribute to the observed variability in suppression thresholds between ON and OFF fibres.

## Discussion

### Selective suppression based on optic nerve’s fibre diameter

This study demonstrates that frequency-induced neuromodulation (FIN) can suppress action potential conduction in optic nerve fibres in a diameter-dependent manner. Suppression thresholds were found, where consistent maximal suppression was found even when the amplitude of FIN was increased at high frequency range (> 1000 Hz). These suppression thresholds were found to increase with FIN frequency and decrease with fibre diameter. These findings are consistent with previous reports of high-frequency conduction block in peripheral nerves, shown computationally [16] or in live models [45], and suggest that similar mechanisms may operate in the optic nerve.

Importantly, suppression was not uniform across all frequencies. At lower frequencies (< 500 Hz), suppression was often partial and inconsistent, with spikes reappearing at higher amplitudes. This variability may reflect a mix of mechanisms, including anodal block due to sustained hyperpolarisation and subthreshold modulation of membrane excitability. At the lowest frequency tested (10 Hz), the response at the last node resembled that characteristic of the high-frequency conduction block, with only 1 spike was detected at the most distal node for many spatial locations. However, maximal suppression at this frequency was attributed to a mechanism that is separate from the suppression at higher frequency range. The block in this frequency range was also shown to be inconsistent, and that it significantly altered the spike timing of the membrane upstream of the optic nerve fibre.

Our findings at the lower frequency range below 500 Hz also supported previous experimental findings, where the sinusoidal stimuli of 25–150 Hz were efficacious in activating retinal [12] and hippocampal cells [13]. Our results showed minimum suppression probability around the same frequency range, which could be associated with maximum excitability of the fibres, making suppression unlikely. In [13], using a waveform of 50 Hz at a stimulus amplitude of 30 µA could produce selective activation of interneurons in rat hippocampus, while avoiding activations of larger pyramidal cells. We did not observe prominent size-based differences at 50 Hz, but at 25 Hz. This shift in frequency could be caused by the difference in morphology or biophysical properties of optic nerve fibres compared to hippocampal neurons.

At the intermediate frequency range (500–750 Hz), a transitional zone was exhibited, where maximal suppression became more likely but remained sensitive to fibre diameter. Notably, larger fibres (4.3 μm) required higher amplitudes for suppression and were more prone to spike reappearance, indicating a reversal in diameter-dependent effects. This could potentially explain previous in vivo findings of CNS blocks at frequency range of around hundreds of Hz [10].

### Mechanisms of suppression in the optic nerve

The model revealed distinct suppression behaviours across frequency bands. At low frequencies (e.g., 25 Hz), suppressions were mostly partial, and likely to represent a mix of mechanisms. The high likelihood of maximal suppression at 10 Hz, which decreased with increasing frequency up to around 500 Hz, an anodal or cathodal block was mostly responsible for the reduction in spike numbers [15]. The hyperpolarisation from the interference electrode also altered the upstream response either due to direct modulation of FIN, or the secondary suppression due to the antidromic passage of spikes originating from the optic nerve nodes, disrupting the passage of the orthodromic action potential spikes. While effort has been made to minimise the effect of FIN on the cell body by placing the FIN electrode at least 5 mm away from the centre of the AIS, longitudinal spread of the electric current or antidromic propagation could still reach the cell body from the FIN site.

As frequency increased, the charge per cycle decreased, weakening the anodal block and allowing for another mechanism of suppression. At high frequencies (≥ 1000 Hz), suppression became robust across all fibres tested. The suppression threshold $\:{th}_{sup}$ was also minimally altered by the change in the z-shift or the temporal shift between the retinal stimulation and the FIN current at this frequency range, in comparison to the lower frequencies tested (see Fig. 1 of Supplementary Information 5 and Fig. 1 of Supplementary Information 6). Due to the similarity in characteristics with the conduction block previously reported in PNS [16, 46, 47], it could be inferred that the same type of block can happen in the optic nerve. Bhadra et al. showed that this type of suppression is quickly reversible, and thus does not involve any injury to the nerve to cause suppression. Rather, it was linked to the activity of the fast sodium channel in the optic nerve fibre, as shown by Ackermann et al. [47]. Our test that involved altering the maximal conductance of the sodium channels near the cell body also supported the hypothesis that the suppression at high frequencies is affected by the excitability of the cells.

Excitation was also observed in specific conditions, and often near the suppression thresholds at high frequency bands. For example, at 4000 Hz and 80 µA, ON fibres exhibited increased spiking before suppression took hold. This suggests that FIN can enhance excitability when the FIN amplitude is below the suppression threshold.

### Selective suppression based on the cell type

Attempts to selectively suppress ON or OFF fibres revealed only minor differences in suppression thresholds, typically within a narrow amplitude window (~ 5 µA). These differences were inconsistent across fibre diameters and frequencies, but more commonly found in the largest fibre tested. The small amplitude window may limit the practical feasibility of functional selectivity based solely on RGC subtype.

However, upstream membrane properties—particularly sodium channel density near the soma and AIS—were shown to influence suppression thresholds. Modifying these properties in the OFF-fibre model shifted suppression thresholds by up to 5 µA, indicating that intrinsic excitability plays a role in FIN responsiveness. While this study only tested two RGC subtypes, other morphological and biophysical variations in RGC have been reported [48, 49], which could potentially amplify the difference in suppression threshold and improve the practical feasibility of selective suppression based on the cell type.

### Implications for bionic eye systems

The ability to suppress action potentials based on fibre diameter offers a potential strategy for improving selectivity in visual neuroprostheses. Our results suggested that suppressing action potential selectively based on the fibre diameter could be feasible due to the discrepancy in the suppression thresholds between the smallest fibre and the medium or large fibres. However, several challenges that limit its applicability remain. In particular, the challenges are associated with the distribution of fibre diameters in the optic nerve, the correlation between size and function, as well as the additional spikes generated by FIN.

In rats, more than 90% of the optic nerve fibres are categorised as small fibres. In primates, a spectrum of fibre sizes exists, but small fibres predominate. Similarly, humans’ optic nerve fibre diameters range from 0.1 to 8.3 μm, but its mean diameter is 1 μm [50]. Although the current results showed that differential suppression between the small and large fibres is possible, it may limit the practical application of size-based selective suppression. It is of interest to investigate whether differential suppression based on fibre size is still possible within the small fibre population. Moreover, the relationship between fibre size and the function of RGC needs to be elucidated. While correlations between fibre size groups and RGC functions in some species, such as cats, have been shown [9], the same correlation in other RGC types or species, especially in humans, remains to be investigated.

Relatedly, the small fibres are distributed uniformly in many species. Thus, selectively suppressing one group of fibre diameter across the whole optic nerve would not be useful, as the suppressed fibres can originate from any portion of the retina, and thus FIN modulation could possibly suppress intended signal. This highlights the need for some spatial selectivity and better understanding of the spatial mapping of the optic nerve fibres with regards to the visual field.

Hence, fibre size-based modulation can be combined with other techniques, such as current shaping [51]. In essence, current shaping aims to more precisely control the size and location of the tissue area affected by electrical modulation, thus more closely targeting the neural population of interest and reducing extraneous activation of neighbouring tissue. This is plausibly achieved by adjusting the electrode array design and current delivery method [51].

This ties to the importance of the electrode shape and size on the effects of FIN. This study used planar conductors as the FIN electrodes, spanning across multiple fibre nodes. This configuration resulted in a broader spread of electric potential in comparison to a localised, high voltage area that would result from small electrodes simulated as a point source in previous simulation studies [15, 46]. Here, the use of a larger electrode results in simultaneous modulation of responses at multiple nodes, including more proximal nodes that are closer to the RGC’s cell body. Although the electrical field may not act directly on the cell body, the modulation at these proximal nodes—through electrical coupling with the unmyelinated membrane and potential antidromic spike interactions—increases the likelihood of upstream response modulation that leads to the observed difference between ON and OFF fibres. The present results therefore underscore the importance of electrode size, shape, and placement on the efficacy of FIN, and motivate future work to systematically explore how these parameters can be optimised to enhance the selective inhibition.

Lastly, a drawback of this approach is related to the single spike that was always detected at the start of FIN application in all cases, likely due to the gradual change in the extracellular potential. In practice, this initial activation should be avoided to prevent sending obfuscating information to the brain. This can be achieved by modifying the way to deliver FIN modulation, for example, by starting FIN with a smaller amplitude to avoid activation and slowly increasing it to the required suppressive current. This idea has been shown computationally by Yi and Grill to enable elimination of the single spike at the onset of FIN [52]. Alternatively, FIN can be applied continuously as a tonic modulation, thus the excitation only happens once at the start of the FIN application, and not at the subsequent instances of retinal stimulation.

### Limitations

A major limitation in this computational model is that the myelinated axon fibre part of the ON and OFF fibre models used the same formulation. Recent studies have revealed existing differences in the cell body’s morphology and ion channel kinetics of RGCs that could be associated to their function [48]. However, variations in the myelinated axon region between different types of RGCs are rarely studied. Besides this, the myelinated fibre model only included the ion channels in the nodal regions, because ion channels at the other fibre regions have not been extensively investigated or validated [43]. It is likely that any change to the fibre’s membrane properties would change its response to FIN, and thus this information is essential to better assess the capability of FIN in achieving functionally-selective suppression.

Another important consideration is that the model was formulated using data from rats, and also validated using recordings from rat neurons. Hence, the translatability of the results to other species may be limited, due to the differences in the fibre size distributions and optic nerve geometry.

The finite element model simplifies the anatomy of the eye and optic nerve by assuming perfect insulation at the outermost boundaries and neglecting the conductivity of surrounding tissues. Within the optic nerve, multiple anatomical layers were represented from the nerve fibre space to the surrounding fat tissue, whereas the eye was modelled as a homogeneous bulk medium with the conductivity of the retina. Other electrically conductive tissues external to the eye and optic nerve, such as muscle, blood vessels, and bone, were not explicitly modelled.

Although the inclusion of a conductive exterior medium would alter the electric potential distribution, this modelling choice was made for computational efficiency and to limit confounding effects arising from uncertainties in the geometry and electrical properties of surrounding tissues on the electrical behaviour within the nerve fibre space. Importantly, the aim of this study was to investigate qualitative trends in suppression as a function of fibre diameter rather than absolute stimulation thresholds. Consistent with this objective, additional simulations incorporating bone as the surrounding medium resulted in measurable quantitative differences in suppression probability, while preserving the main qualitative trends with respect to FIN frequency (Supplementary Fig. 1).

Note also that the simulations were limited to 50 ms, which naturally restricted the maximum number of spikes detected. This choice was made for computational feasibility and to allow exploration across fibre locations and multiple FIN parameter combinations. Longer simulations may yield more robust suppression probability estimates, but they did not alter the qualitative trends. At high FIN frequencies (> 500 Hz), suppression persisted for the duration of FIN delivery, so extending the window added no new information. At lower frequencies (e.g., 10 Hz), the 50 ms window captured only part of the full FIN cycle; however, by simulating multiple FIN phase shifts, the effects of both cathodic and anodic phases could be examined, ensuring that the suppression trends could still be comprehensively assessed.

## Conclusions

This study presents a computational framework for evaluating the suppression of optic nerve fibres using frequency-induced neuromodulation. The results show that FIN can effectively suppress action potential conduction in a diameter-dependent manner, with high-frequency stimulation (≥ 1000 Hz) yielding the most consistent suppression across fibre types. While differences in suppression thresholds between ON and OFF fibres were observed, which was influenced by the properties of the sodium channels near the cell body, these differences were small and unlikely to support robust functional selectivity. Studying more extreme morphological or biophysical differences between cell types may result in a better separation of suppression thresholds at high frequency bands. Suppression at lower frequencies was generally partial and involved complex interactions with upstream membrane dynamics. These findings highlight the importance of considering both geometric and biophysical properties when designing stimulation protocols for visual prostheses.

Overall, FIN offers an interesting avenue for enhancing selectivity in bionic eye systems, particularly when integrated with complementary techniques. Future work should focus on validating these findings in vivo, exploring the role of fibre type diversity, and refining stimulation strategies to minimise unintended activations.

## Supplementary Information

Below is the link to the electronic supplementary material.

Supplementary Material 1 (DOCX 21.4 KB)

Supplementary Material 2 (DOCX 18.9 KB)

Supplementary Material 3 (DOCX 17.1 KB)

Supplementary Material 4 (DOCX 1.06 MB)

Supplementary Material 5 (DOCX 364 KB)

Supplementary Material 6 (DOCX 925 KB)

Supplementary Material 7 (PDF 471 KB)
