# Supplementary material for: Computational modelling of the suppression of optic nerve fibre
Source: Med Biol Eng Comput. 2026 Feb 23;64(4):1441–56. doi: 10.1007/s11517-026-03541-z (PMC13121198; doi:10.1007/s11517-026-03541-z)
Supplement: Supplementary file 1 — Supplementary Material 1 (DOCX 21.4 KB) [file 11517_2026_3541_MOESM1_ESM.docx]

Article title: Computational modelling of the suppression of optic nerve fibre

Journal name: Medical and Biological Engineering and Computing

Authors:

Ariastity Pratiwi^1,2^, Orsolya Kekesi^2^, Alejandro Barriga-Rivera^1,2^, and Gregg Suaning^2,3^

^1^ Department of Applied Physics III, University of Seville, Seville, Spain

^2^ School of Biomedical Engineering, University of Sydney, Sydney, NSW, Australia

^3^ Freiburg Institute for Advanced Studies, University of Freiburg, Freiburg, Germany

Corresponding author: Ariastity Pratiwi ([apratiwi@us.es](mailto:apratiwi@us.es))

**Supplementary Information 2: The geometrical and electrical parameters of the ON and OFF RGCs, modified from Guo, et al [1].**

|  | | **Dendrite** | **Soma** | **AH** | **AIS** | **DA** |
| --- | --- | --- | --- | --- | --- | --- |
| **Average diameter (µm)** | | 2.4 | 28 | 0.9 | 0.8 | 0.4 |
| **Length (µm)** | | 66 | 20 | 50 | 53 | 265 |
| **Number of segments** | | 11 | 51 | 31 | 31 | 31 |
| **Intracellular axial resistivity (Ω.cm)** | | 110 | 110 | 110 | 110 | 110 |
| **Specific membrane capacitance (µF/cm^2^)** | | 1 | 1 | 1 | 1 | 1 |
| $g_{\mathrm{Na}}$ **(S/cm^2^)** | **ON** | 50.2e-3* | 112.7e-3* | 94.1e-3* | 220.9e-3* | 110.8e-3* |
|  | **OFF** | 38.7e-3* | 128.6e-3* | 94.1e-3* | 201.9e-3* | 80.5e-3* |
| $g_{\mathrm{Kdr}}$ **(S/cm^2^)** | **ON** | 41.3e-3* | 39.5e-3* | 33.8e-3* | 43.2e-3* | 67.5e-3* |
|  | **OFF** | 41.3e-3* | 39.5e-3* | 33.8e-3* | 43.2e-3* | 67.5e-3* |
| $g_{\mathrm{Ka}}$ **(S/cm^2^)** | **ON** | 13.9e-3 | 18.9e-3 | 18.9e-3 | 18.9e-3 | - |
|  | **OFF** | 13.9e-3 | 18.9e-3 | 18.9e-3 | 18.9e-3 | - |
| $g_{\mathrm{KCa}}$ **(S/cm^2^)** | **ON** | 1.7e-3* | 3.27e-3* | 4.02e-3* | 4.23e-3* | 2.94e-3* |
|  | **OFF** | 1.7e-3* | 3.27e-3* | 4.02e-3* | 4.23e-3* | 2.94e-3* |
| $g_{\mathrm{Ca}}$ **(S/cm^2^)** | **ON** | 2.1e-3 | 1.6e-3 | 1.6e-3 | 1.6e-3 | - |
|  | **OFF** | 2.1e-3 | 1.6e-3 | 1.6e-3 | 1.6e-3 | - |
| $g_{\mathrm{HCN}}$ **(S/cm^2^)** | **ON** | 0.0572e-3 | 0.0286e-3 | 0.0286e-3 | 0.0286e-3 | 0.0286e-3 |
|  | **OFF** | 4.2e-3 | 2.1e-3 | 2.1e-3 | 2.1e-3 | 2.1e-3 |
| $g_{l}$ **(S/cm^2^)** | **ON** | 0.259e-3 | 0.259e-3 | 0.259e-3 | 0.259e-3 | 0.259e-3 |
|  | **OFF** | 0.0519e-3 | 0.0519e-3 | 0.0519e-3 | 0.0519e-3 | 0.0519e-3 |
| $g_{\mathrm{CaT}}$ **(S/cm^2^) (OFF-only)** | | 0.9915e-3 | 0.1983e-3 | 0.1983e-3 | 0.1983e-3 | 0.1983e-3 |

* The value was changed from the original model

| **Ion channel** | **State variable** | $\alpha$ | $\beta$ | **Reversal Potential (mV)** |
| --- | --- | --- | --- | --- |
| Na | m | $\frac{-0.6(V_{n}+30)}{e^{-0.1(V_{n}+30)}-1}$ | $20e^{\frac{-1(V_{n}+55)}{18}}$ | 35 |
|  | h | $0.4e^{\frac{-1(V_{n}+50)}{20}}$ | $\frac{6}{e^{-0.1(V_{n}+20)}+1}$ |  |
| K_dr_ | n | $\frac{-0.02(V_{n}+40)}{e^{-0.1(V_{n}+40)}-1}$ | $0.4e^{\frac{-1(V_{n}+50)}{80}}$ | -68 |
| K_a_ | a | $\frac{-0.003(V_{n}+90)}{e^{-0.1(V_{n}+90)}-1}$ | $0.1e^{\frac{-1(V_{n}+30)}{10}}$ |  |
|  | h_A_ | $0.04e^{\frac{-1(V_{n}+70)}{20}}$ | $\frac{0.6}{e^{-0.1(V_{n}+40)}+1}$ |  |
| K_Ca_ | $I_{K,Ca}=g_{K,Ca}\frac{\left( \frac{\mathrm{Ca}_{i}}{0.001} \right)^{2}}{\left( \frac{\mathrm{Ca}_{i}}{0.001} \right)^{2}+1}\times(V_{n}-E_{K})$ | | |  |
| Ca | c | $\frac{-0.15(V_{n}+13)}{e^{-0.1(V_{n}+13)}-1}$ | $10e^{\frac{-1(V_{n}+38)}{18}}$ | $\frac{\mathrm{RT}}{2F}\ln\left( \frac{1.8}{\mathrm{Ca}_{i}} \right)$ |
| Ca_T_ | m_T_ | $\frac{1}{1.7+ e^{-\frac{(V_{n}+28.8)}{13.5}}}$ | $\frac{1+e^{-\frac{V_{n}+63}{7.8}}}{1.7+e^{-\frac{V_{n}+28.8}{13.5}}}$ | 35 |
|  | h_T_ | $e^{-\frac{V_{n}+160.3}{17.8}}$ | $\alpha_{\mathrm{hT}}(\sqrt{0.25+e^{\frac{V_{n}+83.5}{6.3}}-0.5})$ |  |
|  | d | $\frac{1+e^{\frac{V_{n}+37.4}{30}}}{\left( 240\left( 0.5+\sqrt{0.25+e^{\frac{V_{n}+83.5}{6.3}}} \right) \right)}$ | $\alpha_{d}\sqrt{0.25+e^{\frac{V_{n}+83.5}{6.3}}}$ |  |
| HCN | y | $y_{\infty}=\frac{1}{1+e^{\frac{V_{n}+75}{5.5}}}$ | $\tau_{h}=588.2\frac{e^{0.01(V_{n}+10)}}{1+e^{0.2(V_{n}+10)}}$ | 35 |
| Leak | - | - | - | -70.5 |

**References**

[1] T. Guo *et al.*, “Insights from computational modelling: selective stimulation of retinal ganglion cells,” *Brain and Human Body Modeling 2020*, p. 233, 2021.
