# Supplementary material for: Computational modelling of the suppression of optic nerve fibre
Source: Med Biol Eng Comput. 2026 Feb 23;64(4):1441–56. doi: 10.1007/s11517-026-03541-z (PMC13121198; doi:10.1007/s11517-026-03541-z)
Supplement: Supplementary file 2 — Supplementary Material 2 (DOCX 18.9 KB) [file 11517_2026_3541_MOESM2_ESM.docx]

Article title: Computational modelling of the suppression of optic nerve fibre

Journal name: Medical and Biological Engineering and Computing

Authors:

Ariastity Pratiwi^1,2^, Orsolya Kekesi^2^, Alejandro Barriga-Rivera^1,2^, and Gregg Suaning^2,3^

^1^ Department of Applied Physics III, University of Seville, Seville, Spain

^2^ School of Biomedical Engineering, University of Sydney, Sydney, NSW, Australia

^3^ Freiburg Institute for Advanced Studies, University of Freiburg, Freiburg, Germany

Corresponding author: Ariastity Pratiwi ([apratiwi@us.es](mailto:apratiwi@us.es))

**Supplementary Information 4: The geometrical and electrical parameters of the MAF section of the optic nerve fibre, adapted from Li, et al [1].**

| **Parameter** | **Diameter (µm)** | | |
| --- | --- | --- | --- |
|  | 1.4 | 2.8 | 4.3 |
| Node length (µm) | 1 | 1 | 1 |
| Node diameter (µm) | 0.7 | 1.1 | 1.5 |
| Paranode length (µm) | 3 | 3 | 3 |
| Paranode diameter (µm) | 0.7 | 1.1 | 1.5 |
| Juxtaparanode length (µm) | 7.3 | 15.7 | 27.3 |
| Juxtaparanode diameter (µm) | 1.2 | 2.0 | 2.6 |
| Internode length (µm) | 24.9 | 40.9 | 52.1 |
| Internode diameter (µm) | 1.2 | 2.0 | 2.6 |
| Periaxonal space width (µm) | 0.002 | 0.004 | 0.004 |
| Number of myelin lamellae | 13 | 28 | 40 |
| $g_{Na,f}$ (S.cm^-2^) | 0.75 | | |
| $g_{K,a}$ (S.cm^-2^) | 0.25 | | |
| $g_{Na, p}$ (S.cm^-2^) | 0.001 | | |
| $g_{K,s}$ (S.cm^-2^) | 0.012 | | |
| $g_{K, f}$ (S.cm^-2^) | 0.02 | | |
| $g_{L}$ (S.cm^-2^) | 0.007 | | |
| $E_{\mathrm{Na}}$ (mV) | 48 | | |
| $E_{K}$ (mV) | -98 | | |
| $E_{L}$ (mV) | -90 | | |

| Ion channel | State variable | $\alpha_{x}$ | $\beta_{x}$ | Reversal potential (mV) |
| --- | --- | --- | --- | --- |
| Na_f_ | m | $\frac{1.86(V_{n}+21.4)}{1-e^{(-21.4-V_{n})/10.3}}$ | $\frac{-0.086(V_{n}+25.7)}{1-e^{(V_{n}+25.7)/9.16}}$ | 50 |
|  | h | $\frac{-0.062(V_{n}+114)}{1-e^{(V_{n}+114)/11}}$ | $\frac{2.3}{1+e^{(-31.8-V_{n})/13.4}}$ |  |
| K_a_ | a | $\frac{0.006(V_{n}+90)}{1-e^{(-90-V_{n})/10}}$ | $0.1e^{(-30-V_{n})/10}$ | -90 |
|  | b | $0.004e^{(-70-V_{n})/20}$ | $\frac{0.6}{1+e^{(-40-V_{n})/10}}$ |  |
| Na_p_ | p | $\frac{0.01(V_{n}+27)}{1-e^{(-27-V_{n})/10.2}}$ | $\frac{0.00025(V+34)}{1-e^{(V+34)/10.2}}$ | 50 |
| K_s_ | s | $\frac{0.3}{1-e^{(-27-V_{n})/5}}$ | $\frac{-0.03}{{1+e}^{(-10-V_{n})}}$ | -90 |
| K_f_ | n | $\frac{0.00462(V_{n}+83.2)}{1-e^{(-83.2-V_{n})/1.1}}$ | $\frac{-0.0824(V_{n}+66)}{1-e^{(V_{n}+66)/10.5}}$ | -90 |
| Leak | - | - | - | -72 |

**References**

[1] M. Li *et al.*, “A simulation of current focusing and steering with penetrating optic nerve electrodes,” *Journal of neural engineering*, vol. 10, no. 6, p. 066007, 2013.
