# Supplementary material for: Computational modelling of the suppression of optic nerve fibre
Source: Med Biol Eng Comput. 2026 Feb 23;64(4):1441–56. doi: 10.1007/s11517-026-03541-z (PMC13121198; doi:10.1007/s11517-026-03541-z)
Supplement: Supplementary file 3 — Supplementary Material 3 (DOCX 17.1 KB) [file 11517_2026_3541_MOESM3_ESM.docx]

Article title: Computational modelling of the suppression of optic nerve fibre

Journal name: Medical and Biological Engineering and Computing

Authors:

Ariastity Pratiwi^1,2^, Orsolya Kekesi^2^, Alejandro Barriga-Rivera^1,2^, and Gregg Suaning^2,3^

^1^ Department of Applied Physics III, University of Seville, Seville, Spain

^2^ School of Biomedical Engineering, University of Sydney, Sydney, NSW, Australia

^3^ Freiburg Institute for Advanced Studies, University of Freiburg, Freiburg, Germany

Corresponding author: Ariastity Pratiwi ([apratiwi@us.es](mailto:apratiwi@us.es))

**Supplementary Information 1: The geometrical and electrical parameters of the retina and optic nerve tissue.**

|  | **Thickness (µm)** | **Conductivity (S.m-1)** |
| --- | --- | --- |
| Epidural fat | 200 | 0.04 [1] |
| Dura mater | 0.36 [1] | 0.065 [1] |
| CSF | 3.1 [1] | 1.7 [1] |
| Pia mater | 0.01 [1][2] | 0.016 [1][2] |
| Fibre space | 160 (radius) [3] | 0.6 (longitudinal), 0.083 (transverse) [3] |
| Retina | - | 0.7 [4] |

**References**

[1] L. Manola, J. Holsheimer, P. Veltink, and others, “Modelling motor cortex stimulation for chronic pain control: electrical potential field, activating functions and responses of simple nerve fibre models,” *Med. Biol. Eng. Comput*, vol. 43, no. 3, pp. 335–343, 2005.

[2] M. Oozeer and others, “A model of the mammalian optic nerve fibre based on experimental data,” *Vis. Res*, vol. 46, no. 16, pp. 2513–2524, 2006.

[3] J. J. Struijk, J. Holsheimer, B. van Veen, and H. Boom, “Paresthesia thresholds in spinal cord stimulation: a comparison of theoretical results with clinical data,” *IEEE Trans. Rehabil. Eng*, vol. 1, no. 2, pp. 101–108, 1993.

[4] S. Lee, H. Kim, S. Park, and others, “In-vivo estimation of tissue electrical conductivities of a rabbit eye for precise simulation of electric field distributions during ocular iontophoresis,” *Int. J. Numer. Methods Biomed. Eng*, vol. 38, no. 1, p. e3540, 2022.
