# Supplementary material for: Computational modelling of the suppression of optic nerve fibre
Source: Med Biol Eng Comput. 2026 Feb 23;64(4):1441–56. doi: 10.1007/s11517-026-03541-z (PMC13121198; doi:10.1007/s11517-026-03541-z)
Supplement: Supplementary file 4 — Supplementary Material 4 (DOCX 1.06 MB) [file 11517_2026_3541_MOESM4_ESM.docx]

Article title: Computational modelling of the suppression of optic nerve fibre

Journal name: Medical and Biological Engineering and Computing

Authors:

Ariastity Pratiwi^1,2^, Orsolya Kekesi^2^, Alejandro Barriga-Rivera^1,2^, and Gregg Suaning^2,3^

^1^ Department of Applied Physics III, University of Seville, Seville, Spain

^2^ School of Biomedical Engineering, University of Sydney, Sydney, NSW, Australia

^3^ Freiburg Institute for Advanced Studies, University of Freiburg, Freiburg, Germany

Corresponding author: Ariastity Pratiwi ([apratiwi@us.es](mailto:apratiwi@us.es))

**Supplementary Information 3: Optimisation of the combined RGC-optic nerve models.**

The *in vitro* physiological data used to fit the combined RGC-optic nerve models were obtained from Hadjinicolaou et al [1] and Foster et al [2]. The objective of this procedure is to modify the conductance values, within the known biological boundaries, so that the response of the fibre models closely resembles the responses shown *in vitro.* The metrics used to measure closeness were the frequency response of an RGC when given intracellular sinusoidal stimulus of various frequencies [1] and the conduction velocity of the optic nerve [2].

The conductance values changed were g_Na_, g_K,dr_, and g_K,Ca_ of the soma, dendrite, AH, AIS, and non-myelinated distal axon. To automatically modify these conductance values, a Markov Chain Monte Carlo (MCMC) method [3] with a Metropolis-Hastings algorithm [4][5] was used. MCMC estimates the value of a parameter by iteratively drawing samples of conductance values from a normal distribution centred around the initial conductance values from Guo et al [6]. Then, the ‘likelihood’ of the sample value (i.e., the probability of the sample values being the correct values to replicate the physiological data) was determined based on a loss function shown in Equation (1) [3]. The likelihood of the current sample set, and the initial sample set were then compared, and the current sample set would become the new initial sample set only if its likelihood was higher than the initial sample set. Thus, if the current sample set was accepted, the subsequent iteration of the MCMC algorithm would choose a sample set that was closer to the actual values, minimising the loss function in each accepted iteration. The process is repeated until the model produced the expected response, as shown by the referenced physiological recordings, or until the maximum number of iterations was reached (set to 200 iterations).

$$Loss=1000 \times\left| {Sample}_{F}-{Reference}_{F} \right|+ 1000 \times\left| {Sample}_{DC}-{Reference}_{DC} \right| (1)$$

Sample_F_ and Reference_F_ were the mean spike frequency produced by the current sample set and the referenced spiking response, respectively. Sample_DC_ and Reference_DC_ were the duty cycle, or the ratio of the time the neuron is spiking and the time the neuron is not spiking per a given period of time. The aggregate loss function was calculated for each iteration, meaning that the loss function of the model cell at each stimulus frequency was summated.

In the referenced experiments, Hadjinicolaou et al subjected rat RGCs to intracellular sinusoidal current input with frequencies ranging from 1-60 Hz. This experiment reported that ON and OFF exhibited an increase in the spiking frequency with the stimulus frequency, and then a decrease as the stimulus frequency was increased further. OFF-A2 RGCs showed lower spiking frequencies than the ON-A2 RGCs [2]. Our modified models demonstrated similar response characteristics when the current input was delivered to the soma of the cells (Fig. 1a-b). The ON fibre model spiked with 1 or more spikes per stimulus period when given a 10 Hz stimulus. Increasing the stimulus frequency further reduced the spike number per period, as described in the experimental study. This also contributed to the reduced spiking frequency with increasing stimulus frequency in Fig. 1b. The response of the ON fibre peaked at 30 Hz before decreasing at higher stimulus frequencies, while the current OFF fibre model showed this peak at 45 Hz. Similarly, in vitro measurements showed the OFF RGC’s peak was at 15 Hz, while the OFF RGC model peaked at 30 Hz. The spiking frequency of the OFF-fibre model was always lower than the ON fibre model at stimulus frequencies higher than 30 Hz. To produce these responses, the current amplitudes used for the ON fibre model were 0.9, 0.9, 0.99, 1.1, and 1.27 nA for stimulus frequencies of 10, 25, 35, 45, and 60 Hz. The current amplitudes used for the OFF-fibre model were 0.4, 0.6, 0.7, 0.9, and 1.27 nA for stimulus frequencies of 10, 25, 35, 45, and 60 Hz.


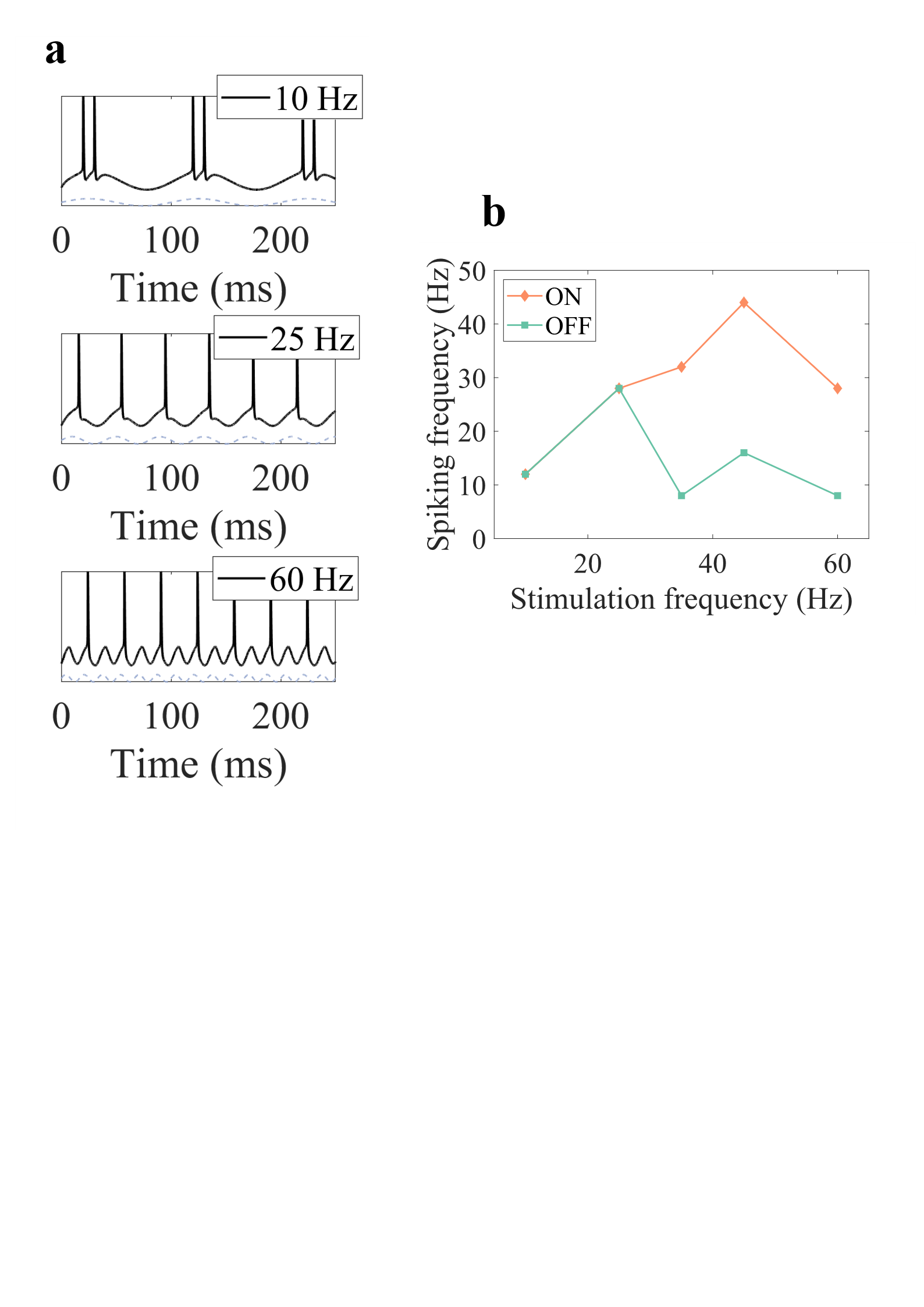


**Fig. 1** (a) The transmembrane potential for the ON fibre model ($d_{f}$= 1.4 µm) when given stimulus frequencies of 10, 25, and 60 Hz. The reduction in the number of spikes per stimulus period was as described in Hadjinicolaou, et al. (b) The spiking frequency of the ON and OFF fibre models plotted against stimulus frequency. The spiking frequency increased with the stimulus frequency for both the ON and OFF fibre models, before reaching a peak and decreasing with the stimulus frequency

Next, the conduction velocity of the myelinated axon fibre (MAF) section of the model was measured for all the fibre diameters tested. The conduction velocity of the ON nerve fibre was measured in response to a square, suprathreshold intracellular stimulus pulse (duration = 0.01 ms, amplitude = 2 nA) applied to the first node of the myelinated fibre, so as to produce a single spike. The first and last nodes of the myelinated nerve fibre were used as reference points to for distance measurement, while the time in the velocity equation was taken as the time difference between the occurrence of the action potential peak at the first node and at the last node. The conduction velocities for $d_{f}$= 1.4, 2.8 and 4.3 μm were 2.5, 4.7, and 5.8 m·s-1, respectively, as seen in Fig. 2 The conduction velocities of the optic nerve fibre models were within the range reported by Foster, et al, of 3 - 20 m/s, for adult rats. The curve of the conduction velocity against the fibre diameter could also be fitted to a linear function, showing the expected increase in conduction velocity with fibre diameter, as described by Sumitomo, et al [7].


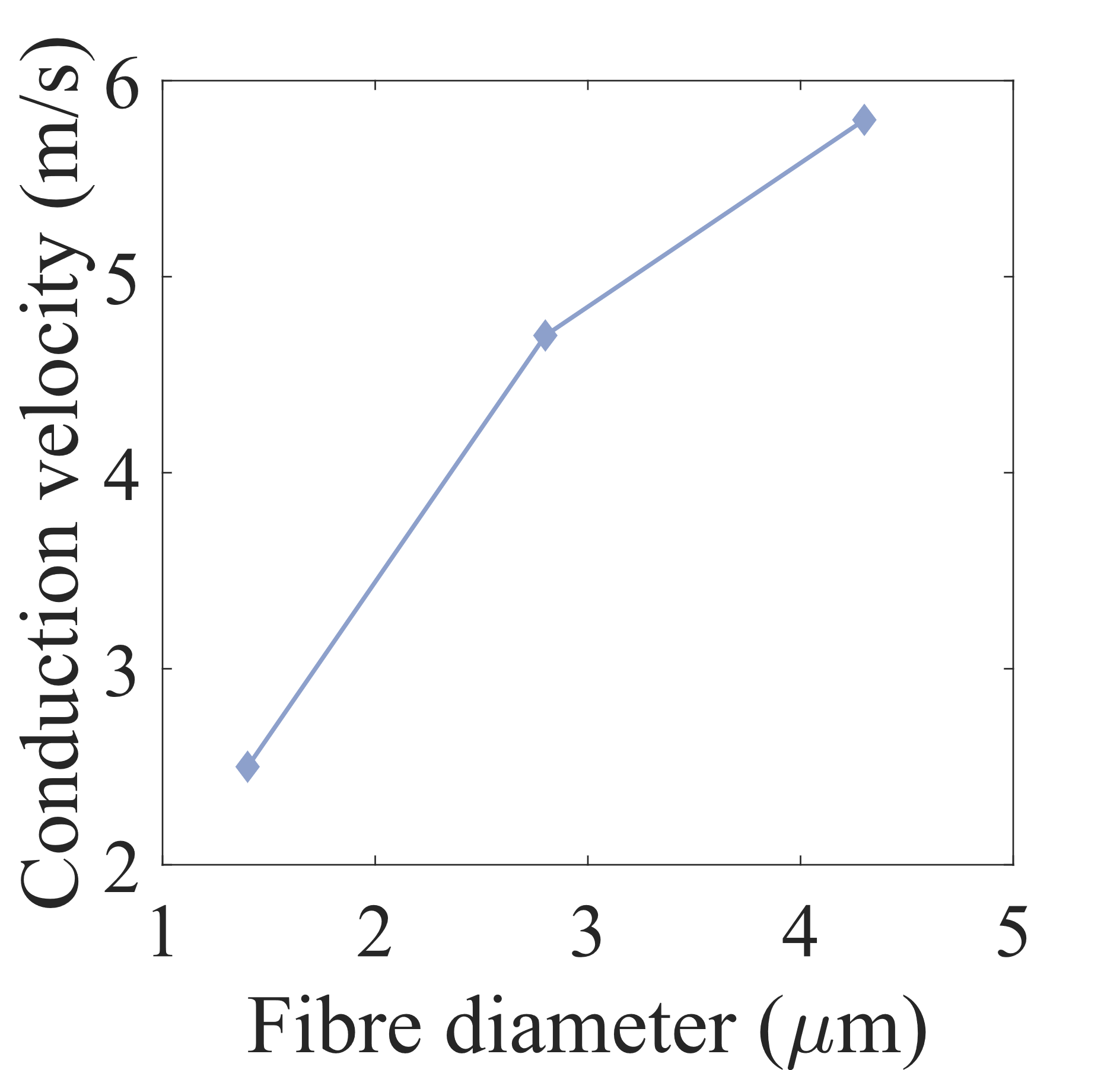


**Fig. 2**. The conduction velocity of the MAF section of the combined RGC-optic nerve model for $d_{f}$= 1.4, 2.8 and 4.3 μm. The conduction velocities could be fitted to a linear function $y= 1.13x+1.12$

As the final model combined two separate sets of ion channel formulations and membrane properties, the behaviour around the junction between the unmyelinated RGC section and the myelinated optic nerve section was examined to ensure that the combination did not produce unpredictable membrane activities. In Fig. 3, the membrane potential at the distal end of the DA section and at the most proximal compartment of the first node are shown at different time points, representing the membrane potentials at the proximal and distal sides of the junction. Here, no FIN was applied and only the spiking activities from the retinal stimulation were present. The electrical coupling between the distal end of the DA and the first node resulted in the expected uniformity of the membrane potential. A drop in voltage was observed in the first compartment distal to the first node, which is the first paranode. The drop in voltage was caused by the differences in membrane capacitance and resistance. Then, the second node showed the spikes with the peak height slightly lower than those at the first node, but higher than at the paranode. This behaviour is expected, as the spikes from the AIS were all transmitted to the second node, but with the lower peak height due to the axial resistance.


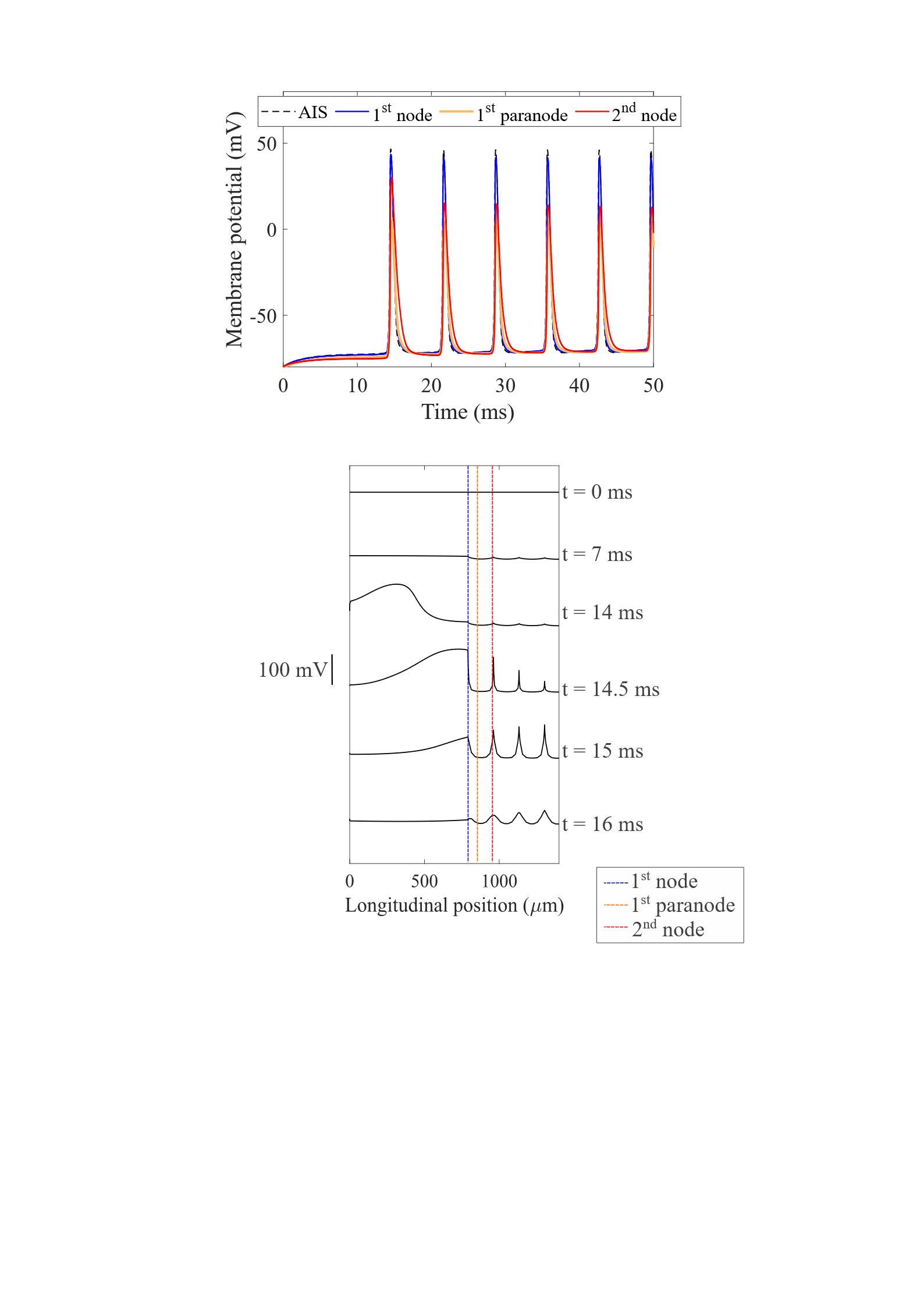


**Fig. 3**. (Top) The membrane potentials of four different reference points along the fibre, which are the AIS, the first node, the first paranode, and the second node, throughout the whole simulation period. (bottom) The membrane potential of a section of the fibre, showing the first node, first paranode, and the second node, at different time points. All spiking activity that began near the soma was conducted along the optic nerve fibre, with spikes occurring at nodes and voltage drops occurring at the paranodes.

**References**

[1] A. E. Hadjinicolaou, S. L. Cloherty, Y.-S. Hung, T. Kameneva, and M. R. Ibbotson, “Frequency responses of rat retinal ganglion cells,” *PLoS One*, vol. 11, no. 6, p. e0157676, 2016.

[2] R. E. Foster, B. W. Connors, and S. G. Waxman, “Rat optic nerve: electrophysiological, pharmacological and anatomical studies during development,” *Developmental Brain Research*, vol. 3, no. 3, pp. 371–386, 1982.

[3] Y. C. Wang *et al.*, “Multimodal parameter spaces of a complex multi-channel neuron model,” *Frontiers in Systems Neuroscience*, vol. 16, p. 999531, 2022.

[4] N. Metropolis, A. Rosenbluth, M. Rosenbluth, A. Teller, and E. Teller, “Perspective on ‘Equation of state calculations by fast computing machines,’” *J. Chem. Phys*, vol. 21, pp. 1087–1092, 1953.
[5] W. K. Hastings, “Monte Carlo sampling methods using Markov chains and their applications,” 1970.

[6] T. Guo *et al.*, “Insights from computational modelling: selective stimulation of retinal ganglion cells,” *Brain and Human Body Modeling 2020*, p. 233, 2021.

[7] I. Sumitomo, K. Ide, K. Iwama, and T. Arikuni, “Conduction velocity of optic nerve fibers innervating lateral geniculate body and superior colliculus in the rat,” Experimental Neurology, vol. 25, no. 3, pp. 378–392, Nov. 1969, doi: 10.1016/0014-4886(69)90132-0. Available: <https://doi.org/10.1016/0014-4886(69)90132-0>
