# Supplementary material for: Computational modelling of the suppression of optic nerve fibre
Source: Med Biol Eng Comput. 2026 Feb 23;64(4):1441–56. doi: 10.1007/s11517-026-03541-z (PMC13121198; doi:10.1007/s11517-026-03541-z)
Supplement: Supplementary file 5 — Supplementary Material 5 (DOCX 364 KB) [file 11517_2026_3541_MOESM5_ESM.docx]

Article title: Computational modelling of the suppression of optic nerve fibre

Journal name: Medical and Biological Engineering and Computing

Authors:

Ariastity Pratiwi^1,2^, Orsolya Kekesi^2^, Alejandro Barriga-Rivera^1,2^, and Gregg Suaning^2,3^

^1^ Department of Applied Physics III, University of Seville, Seville, Spain

^2^ School of Biomedical Engineering, University of Sydney, Sydney, NSW, Australia

^3^ Freiburg Institute for Advanced Studies, University of Freiburg, Freiburg, Germany

Corresponding author: Ariastity Pratiwi ([apratiwi@us.es](mailto:apratiwi@us.es))

**Supplementary Information 5: The effects of z-shift on the fibre’s response to FIN**

The results presented in Fig. 4a of the main text included the contributions of fibres at different z-shifts. The z-shift represents the longitudinal distance between the centre of the electrode and the centre of the closest node. As it is defined as a ratio of the internodal distance (0, 0.25, and 0.5), the absolute distance represented by the same z-shift value would differ for different fibre diameters. This is because the internodal distance also changes based on the fibre diameter.

The effects of the longitudinal shift on the response of fibre models to FIN are analysed by simulating the ON fibre models at all locations, grouped by the z-shifts. As seen in Fig. 1 below, z-shift could significantly alter the $n_{spike}$ at different FIN frequencies and fibre diameters. However, the effect of z-shift seemed to be non-uniform. Increasing the z-shift (i.e., increasing the longitudinal distance between the electrode and the centre of the node) could have a minimum impact, as shown by all fibre diameters at $f_{Int}=$ 25 Hz (Fig. 1a). This trend continued up to $f_{Int}=$ 100 Hz (result not shown). Increasing the $f_{Int}$ further to 250 Hz resulted in the increase of $n_{spike}$ with the z-shift for some fibres, as shown by $d_{f}=1.4$µm at $f_{Int}=$ 250 Hz (Fig. 1b). However, it could also decrease the $n_{spike}$, as shown by $d_{f}=1.4$µm at $f_{Int}=$ 550 Hz (Fig. 1c). At frequencies > 1000 Hz, z-shift showed a minimized effect on the $n_{spike}$ for all fibre diameters, exemplified by the little to no gradient of $n_{spike}$ with z-shift for all fibre diameters at $f_{Int}=$ 5000 Hz (Fig. 1d). The variable effects of z-shift, together with its dependence on fibre diameter and FIN frequencies, highlight the importance of considering these variations in the calculation of the suppression probability per fibre.


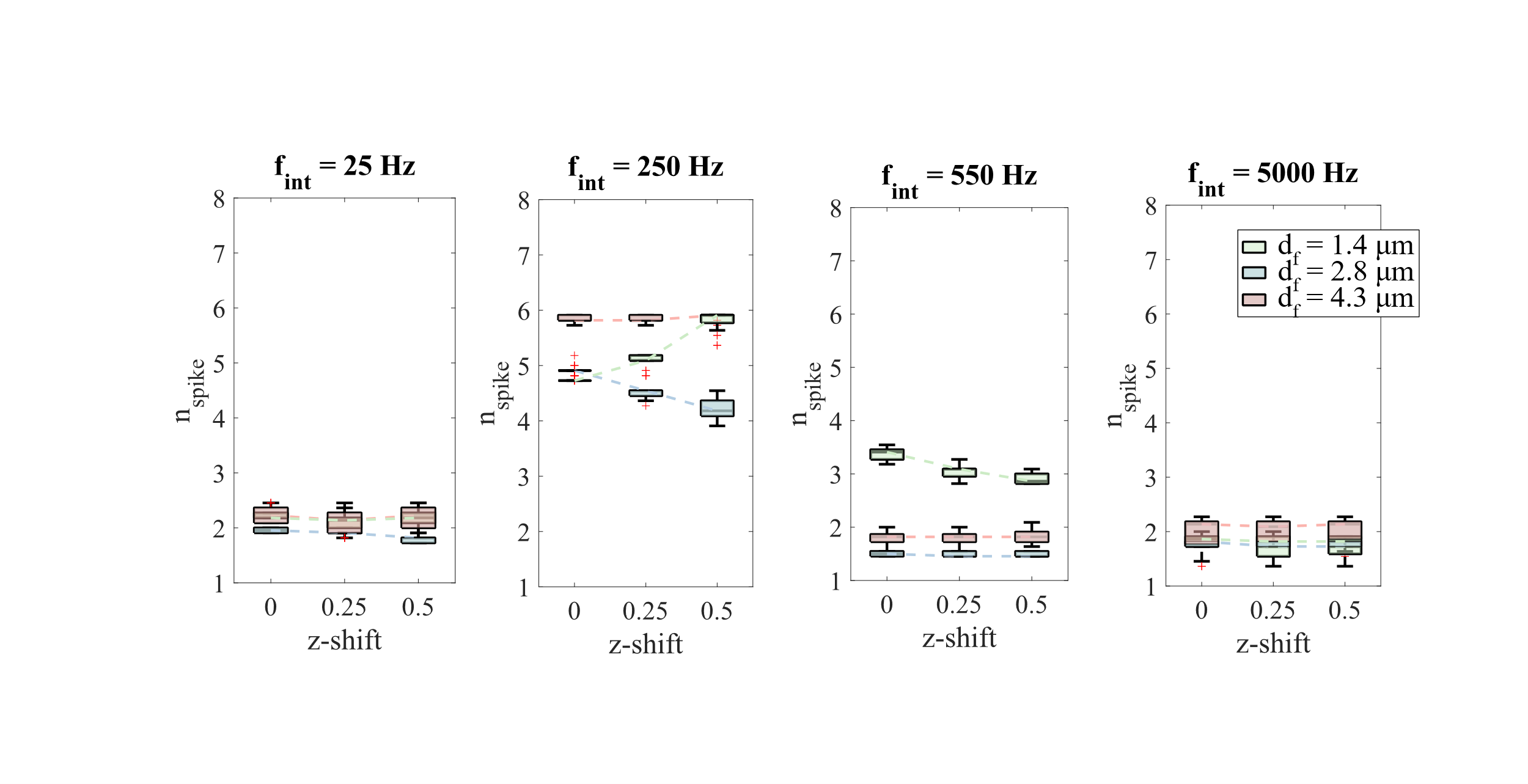


**Fig.1**. The boxplots of z-shift against $n_{spike}$ for ON fibre models at all xy locations, grouped by the fibre diameter $d_{f}$, at (a) $f_{Int}=$ 25 Hz, (b) $f_{Int}=$ 250 Hz, (c) $f_{Int}=$ 550 Hz, and (d) $f_{Int}=$ 5000 Hz. The dashed lines show connected the median value of each z-shift.
