# Supplementary material for: Computational modelling of the suppression of optic nerve fibre
Source: Med Biol Eng Comput. 2026 Feb 23;64(4):1441–56. doi: 10.1007/s11517-026-03541-z (PMC13121198; doi:10.1007/s11517-026-03541-z)
Supplement: Supplementary file 6 — Supplementary Material 6 (DOCX 925 KB) [file 11517_2026_3541_MOESM6_ESM.docx]

Article title: Computational modelling of the suppression of optic nerve fibre

Journal name: Medical and Biological Engineering and Computing

Authors:

Ariastity Pratiwi^1,2^, Orsolya Kekesi^2^, Alejandro Barriga-Rivera^1,2^, and Gregg Suaning^2,3^

^1^ Department of Applied Physics III, University of Seville, Seville, Spain

^2^ School of Biomedical Engineering, University of Sydney, Sydney, NSW, Australia

^3^ Freiburg Institute for Advanced Studies, University of Freiburg, Freiburg, Germany

Corresponding author: Ariastity Pratiwi ([apratiwi@us.es](mailto:apratiwi@us.es))

**Supplementary Information 6: The effects of phase shift on the fibre’s response to FIN**

It is of interest to study the influence of temporal alignment between the start of the FIN and the retinal stimulation pulse. To study this, a supplementary simulation of an ON fibre with $d_{f}=$ 1.4 µm at the base location was conducted, where three temporal alignments between the FIN and the retinal pulse were modelled. The temporal alignment was defined by a phase shift of $\phi$ = 0, $\pi/2$, and $\pi$. Here, $\phi$ = 0 indicates that the start of the retinal stimulation pulse coincides with the start of the anodic (positive current) phase of FIN, $\phi$ = $\pi/2$ indicates that the start of the retinal stimulation pulse coincides with the peak amplitude of the anodic phase, and $\phi$ = $\pi$ indicates that the start of the retinal stimulation pulse coincides with the start of the cathodic (negative current) phase.

Fig. 1 shows that for the modelled fibre, the variation of $n_{spike}$ (spike counts at the last node) with phase shift occurred at $f_{int}$ between 10 and 550 Hz. For higher FIN frequencies, the ${th}_{sup}$ did not change with phase shift, meaning that maximal suppression could be produced regardless of the phase of the FIN current relative to the retinal stimulation.


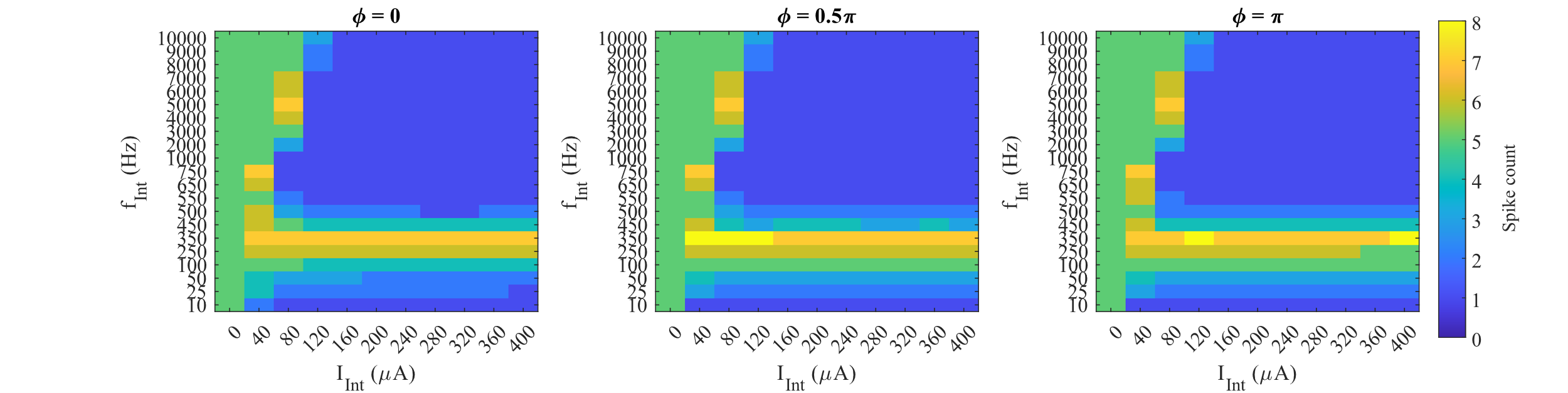


**Fig. 1**. The colour grids showing the spike counts at the most distal node at various FIN parameters, for an ON fibre with $d_{f}=$ 1.4 µm at the base location (x = y = 0). The change in spike counts with the phase shift is shown in the lower frequency range (≤ 550 Hz) only.

The robustness of the maximal suppression at higher FIN frequencies could benefit the practical application of FIN, as suppression could be expected whenever the FIN current is delivered, without having to consider the timing of the retinal spikes. On the other hand, the dependence of the suppression response at the lower FIN frequencies on the phase shift was expected, as the suspected suppression mechanism at this frequency range is the anodal/cathodal block, and hence it is dependent on the total charge delivered at the time of the spiking activity, which in turn is affected by the phase shift.
