# Supplementary material for: Computational modelling of the suppression of optic nerve fibre
Source: Med Biol Eng Comput. 2026 Feb 23;64(4):1441–56. doi: 10.1007/s11517-026-03541-z (PMC13121198; doi:10.1007/s11517-026-03541-z)
Supplement: Supplementary file 7 — Supplementary Material 7 (PDF 471 KB) [file 11517_2026_3541_MOESM7_ESM.pdf]

Article title: Computational modelling of the suppression of optic nerve fibre

Journal name: Medical and Biological Engineering and Computing

Authors:

Ariastity Pratiwi[1,2], Orsolya Kekesi[2], Alejandro Barriga-Rivera[1,2], and Gregg Suaning[2,3]

[1] Department of Applied Physics III, University of Seville, Seville, Spain

[2] School of Biomedical Engineering, University of Sydney, Sydney, NSW, Australia

[3] Freiburg Institute for Advanced Studies, University of Freiburg, Freiburg, Germany

Corresponding author: Ariastity Pratiwi (apratiwi@us.es)

**Supplementary Figure 1: The effects of adding a surrounding medium to the Finite Element Model.**

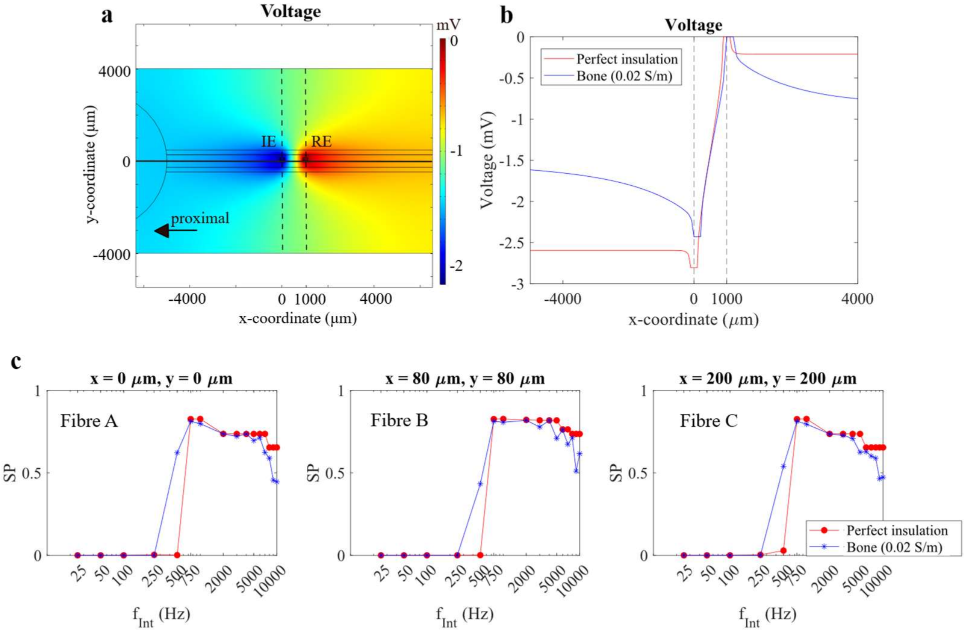


***Supplementary Fig. 1.*** *Comparison of extracellular voltage distributions for simulations assuming an insulating exterior domain versus a surrounding medium with the conductivity of bone. (a) The voltage distribution on a sagittal cut plane of the model, using bone (conductivity value = 0.02 S/m [1]) as an exterior medium, at 1 μA current amplitude. The dashed line labelled IE indicates the location of the interference electrode, while the RE line indicates the reference electrode. The electrical potential was spread throughout the nerve fibre space, up to the bone, with the most negative potential occurring adjacent to the IE. A black horizontal line on the longitudinal axis of the optic nerve indicates a cut line to evaluate the voltage inside the nerve fibre space. (b) The voltage along the cut line in (a), for the model assuming perfect insulation at the boundary of the eye and the optic nerve, and the model including bone surrounding the eye and the optic nerve. Incorporating bone reduced the magnitude of the maximal voltage, as well as the average voltage along the evaluation line, due to an increased spreading of the voltage. (c) The suppression probability (SP) against the frequency of FIN was evaluated for three fibres at different x-y positions. Incorporating bone changed the SP at all fibre locations, with the greatest change occurring at* $f_{Int}$ *= 500 Hz. Besides this, the general trends – negligible SP at* $f_{Int} \leq 250$ *Hz, SP peaking at 750-1000 Hz and gradually decreasing with* $f_{Int}$ *– were maintained.*

## References

[1] S. Gabriel, R. W. Lau, and C. Gabriel, “The dielectric properties of biological tissues: II. Measurements in the frequency range 10 Hz to 20 GHz,” *Phys. Med. Biol.*, vol. 41, no. 11, pp. 2251–2269, Nov. 1996, doi: 10.1088/0031-9155/41/11/002.
